# Short-duration preoperative endocrine therapy alters molecular profiles to predict favourable outcome in ER+/HER2+ early breast cancer: a POETIC translational study

**DOI:** 10.1016/j.ebiom.2025.105823

**Published:** 2025-07-18

**Authors:** Milana Bergamino Sirvén, Elena López-Knowles, Xixuan Zhu, Holly Tovey, Lucy Kilburn, Chris Holcombe, Anthony Skene, John Robertson, Judith M. Bliss, Anastasia Alataki, Ian Smith, Eugene F. Schuster, Mitch Dowsett, Maggie Chon U. Cheang

**Affiliations:** a Clinical Trials and Statistics Unit (ICR-CTSU), The Institute of Cancer Research, London, UK; b Medical Oncology Department, Catalan Institute of Oncology-Badalona, Hospital Germans Trias I Pujol (HGTiP), 08916, Badalona, Spain; c Medical Oncology Department, Hospital Clinic of Barcelona, Barcelona, Spain; d Ralph Lauren Centre for Breast Cancer Research, Royal Marsden Hospital, London, UK; e The Breast Cancer Now Toby Robins Research Centre, The Institute of Cancer Research, London, UK; f Liverpool University Hospitals Foundation Trust, Liverpool, UK; g Royal Bournemouth and Christchurch and Christchurch NHS Foundation Trust, Bournemouth, UK; h Faculty of Medicine & Health Sciences, Queen's Medical Centre, Nottingham, UK; i Royal Marsden Hospital, London, UK

**Keywords:** Breast cancer, HER2+, Aromatase inhibitors, Intrinsic subtypes

## Abstract

**Background:**

About 15–20% of breast cancers (BC) overexpress Human Epidermal Growth Factor Receptor 2 (HER2+), and 50% of them are also oestrogen receptor positive (ER+). Patients with ER+/HER2+ BC with a limited response to systemic therapies are at an increased risk of relapse, thus understanding the mechanisms of resistance is crucial. This study investigates the changes in gene signature expression (ΔGSE) within ER+/HER2+ tumours and their intrinsic subtype (IS) in response to peri-operative aromatase inhibitors (POAI).

**Methods:**

We analysed paired pre-treatment (baseline) and on-treatment (2wk) samples from 313 ER+/HER2+ BC from the POETIC trial using the BC360™ codeset. Early biological response to aromatase inhibitors (AI) was assessed by immunohistochemical Ki67 levels. Association of ΔGSE with biological response was evaluated using T-test and time to recurrence (TTR) with multivariable Cox regression models adjusted for clinicopathological variables.

**Findings:**

The immunity-related signatures were significantly upregulated, while proliferation, *TP53* surrogate mutational status and ER-signalling were downregulated (FDR <0·05) among POAI tumours with low Ki67_2__wk_. In the POAI, 79% (59/75) of Luminal B (LumB) at baseline shifted to Luminal A (LumA) at 2wk and LumA_2__wk_ was associated with better TTR compared to LumB_2__wk_ (HR 0·2; CI 95% 0·06–0·72, p = 0·01). Based on Akaike Information Criterion scores, Ki67 and IS at 2wk provided better fit of the multivariable Cox models over the variables at baseline when predicting TTR.

**Interpretation:**

Assessing on-treatment IS after POAI for ER+HER2+ BC can help to identify a group of low-risk patients with LumA_2__wk_ with good outcomes on de-escalated treatment and patients that require additional treatments.

**Funding:**

10.13039/501100000289Cancer Research UK (CRUK/07/015).

**Research in context**

**Evidence before this study**

Aromatase inhibitors (AIs) are the standard of care and the most effective therapy for postmenopausal women with early oestrogen receptor-positive (ER+) breast cancer (BC). Peri-operative AI (POAI) administration before surgery in high-proliferation ER+/HER2− tumours offers critical insights into early endocrine responsiveness. However, ER+ BC tumours that also overexpress HER2 are highly heterogeneous, with multiple treatment options but variable responses to therapies, including limited antiproliferative response to endocrine treatment. Despite the established role of AIs in ER+ BC, evidence on their specific impact on survival in ER+/HER2+ early BC is limited. Additionally, the role of intrinsic subtypes and their changes in response to POAI remains underexplored. This study addresses this gap by investigating changes in gene signature expression and intrinsic subtype in ER+/HER2+ early BC within the POETIC trial framework.

**Added value of this study**

This study demonstrates that the Luminal A intrinsic subtype status, assessed after two weeks of POAI, predicts favourable outcomes in ER+/HER2+ early BC. These findings suggest that patients achieving a Luminal A status after POAI are candidates for de-escalated treatment. Conversely, patients with other subtypes may require more intensive therapeutic strategies. This approach provides an important perspective on treatment stratification in ER+/HER2+ BC. This study also demonstrates immune-modulating effects of AI treatment in ER+/HER2+ BC, with a significant enrichment of tumour-infiltrating immune cells and immune-related characteristics. AI treatment also leads to the downregulation of key biological processes, including cell proliferation, homologous recombination deficiency, *TP53* surrogate mutational status, and ER-signalling. These changes suggest that AI therapy not only targets oestrogen signalling to inhibit tumour growth but also influences DNA repair mechanisms and potentially reduces tumour aggressiveness, enhancing our understanding of the broader effects of hormonal therapies on tumour biology and immune interactions.

**Implications of all the available evidence**

This study provides important evidence supporting the use of POAI to assess on-treatment intrinsic subtypes in ER+/HER2+ BC. It establishes the rationale for using POAI as a tool to identify a low-risk patient subgroup—those achieving Luminal A status after two weeks of treatment—who are likely to achieve favourable outcomes with de-escalated therapies. It also identifies patients with higher-risk subtypes who may benefit from additional therapeutic interventions, paving the way for more personalised treatment strategies in ER+/HER2+ BC.

## Introduction

Fifteen percent of all breast cancers (BC) overexpress human epidermal growth factor receptor 2 (HER2+), which contributes to molecular heterogeneity and results in different clinical outcomes compared to oestrogen receptor-positive (ER+) HER2-negative (HER2−) disease.^1^, ^2^, ^3^ Beyond anti-HER2 therapy (typically given for one year) and chemotherapy, patients with early ER+/HER2+ generally benefit from adjuvant endocrine therapy, usually Aromatase Inhibitors (AI) in post-menopausal women for 5–10 years, though not all patients respond equally.^4^, ^5^, ^6^ Patients with a limited antiproliferative response to endocrine therapy (ET) remain at a higher risk of recurrence, although anti-HER2 therapies in this subset often mitigate this risk.^3^^,^^7^ However, any residual disease after the anti-HER2 therapy remains at risk of recurrence if the tumour does not respond to AI.^8^^,^^9^

Prior results from the PeriOperative Endocrine Therapy for Individualised Care (POETIC)^7^ trial demonstrated that in ER+/HER2− BC, a simple biomarker, particularly dynamic change in Ki67 following short-duration preoperative AI can predict long-term survival outcome. This suggests its potential utility in personalizing treatment decisions.

Growing evidence supports the notion that intrinsic subtypes (ISs) may offer both predictive and prognostic significance within HER2+ BC, particularly in the context of anti-HER2 therapies.^10^, ^11^, ^12^ Our analysis in POETIC confirmed the hypothesis that pre-treatment HER2-enriched (HER2-E) IS predicts an early poor biological response to AI and a higher risk of relapse in ER+/HER2+ BC when treated with AI alone.^13^ However, despite these insights, the role of perioperative treatment in ER+/HER2+ patients remains insufficiently defined and has not yet been integrated into routine clinical practice.

In this translational study from the POETIC trial, we aim to assess the changes in gene expression profiles following 2 weeks (2wk) of AI therapy and to investigate whether on-treatment molecular characteristics provide superior prognostic value compared to baseline features in ER+/HER2+ BC.

## Methods

### Patients and samples

All ER+/HER2+ BC tumours with available baseline and surgical formalin-fixed paraffin-embedded (FFPE) samples from the POETIC trial were included in this study.^7^ In the POETIC trial patients were randomly assigned (2:1) to POAI (letrozole 2·5 mg per day orally or anastrozole 1 mg per day orally) for 14 days before and following surgery, or no POAI (control).^14^ The 2-week surgical samples are labelled as “on-treatment” in POAI-treated patients and “surgery” in control patients. Diagnostic ER and HER2 statuses were determined locally by immunohistochemistry (IHC), and/or fluorescence in situ hybridization (FISH) for HER2 amplification.

### Immunohistochemistry (IHC) Ki67 analyses

Immunohistochemical Ki67 expression was measured in core biopsies taken at Baseline (Ki67_B_) and in either core or excision biopsies taken at surgery (Ki67_2__wk_), estimated as the percentage of cancer cells staining positive. Ki67 IHC staining was done using the MIB-1 antibody (M7240, DAKO UKP; RRID:AB_2631211).^15^ Ki67 has been validated in our laboratory previously and we are part of the International Ki67 in Breast Cancer Working group. In the control group, Ki67 staining of surgical samples was limited to a randomly selected subset due to the minimal expected change in Ki67 from baseline to surgery.^7^ Early response to AI therapy was defined by residual Ki67 at 2-week timepoint (Ki67_2__wk_), classified as High (≥10%) and Low (<10%) and as a continuous variable. Response categories were further divided into four groups: low–low (Ki67_B_ and Ki67_2__wk_ <10%); high–low (Ki67_B_ ≥10% and Ki67_2__wk_ <10%); high–high (Ki67_B_ and Ki67_2__wk_ ≥10%); and low–high (Ki67_B_ <10%, and Ki67_2__wk_ ≥10%).

### RNA extraction

RNA was extracted from three adjacent macro-dissected 10 μm FFPE sections from the baseline and the surgical block of the patients included in the study. The ROCHE High Pure miRNA isolation kit (Roche, Basel, Switzerland) was used following SOP M027 from The Cancer Genome Atlas (TCGA) Program developed by the Biospecimen Core Resource (BCR) at Nationwide Children's Hospital in Columbus, Ohio. Quantification was done using high-sensitivity RNA Qubit assays (Thermo Fisher Scientific, Carlsbad, CA).

### Gene expression profiling

Gene expression of baseline and on-treatment/surgery samples was assessed using the NanoString nCounter FLEX Platform (Nanostring Technologies, RRID: SCR_023912, Seattle, WA) Breast Cancer 360™ codeset (BC360) according to manufacturer's instructions. The input value was 150 ng of RNA, and raw data was normalised by NanoString according to the BC360 pipeline using 18 house-keeping genes. In our study, the PAM50 assay corresponds to the Research Use Only (RUO) version of the commercial Prosigna test. Additionally, the 46 key biological signatures were analysed using the BC360 codeset, as reported in Supplementary Table S1. Spearman's correlation coefficients (*r*) were calculated between the expressions of the 50 IS genes and proprietary NanoString-based centroids, including LumA, LumB, HER2-E and Basal subtypes.^16^^,^^17^ For each sample, the subtype with the highest *r* was assigned as the IS. The biological signatures were calculated as the median expressions of a group of genes contributing to the same biological pathway/function (Supplementary Table S1). Changes in gene signature expression (ΔGSE) were calculated as the log2 fold change between 2wk and baseline expressions. Changes in IS correlation coefficients were calculated as the differences between 2wk and baseline correlation coefficients. The triple-negative breast cancer signature (TNBC) was excluded due to the lack of biological representation within our cohort.

### Statistics

Statistical analyses were performed using the R software (version 4·3·2). Time to Recurrence (TTR) was defined as the time from randomisation to local, regional, or distant tumour recurrence or death from BC without prior relapse. Second primary cancers and non-breast cancer deaths were censored. Median follow-up times were 5·63 years for POAI and 5·16 years for control, while the number of events for TTR were 23 and 13 for POAI and control groups, respectively. Survival analyses were performed using Kaplan–Meier estimates and Cox proportional hazards models. Multivariable Cox regression models were adjusted for key post-surgical clinicopathological covariates, including tumour size, nodal status, and age. Age was incorporated as a primary covariate given its key role as a surrogate for adjuvant treatment decisions at the time of the study; notably, 65% of patients aged ≥70 years (69/106) did not receive chemotherapy or trastuzumab, compared to only 14% (29/207) of those aged <70 years. This approach was carefully considered to minimise multicollinearity and overlap with other variables. Tumour grade was excluded from the multivariable models due to a limited number of grade 1 cases. Ki67 was analysed as continuous variables in the cox regression models. In Cox regression model, a pair of patients is called concordant (agree) if the risk of the event predicted by the model is lower for the patient whose event came at a later timepoint, and discordant (disagree) if *vice versa*. A pair is tied if two patients have the same predicted risk. The concordance index was calculated as: (agree + tied/2)/(agree + disagree + tied). The Akaike Information Criterion (AIC) is a measure used to assess the relative quality of Cox regression models for a given set of data. It is calculated as: AIC = −2 × log-likelihood +2 × k; where the log-likelihood is the value of the likelihood function for the model, and k represents the number of parameters in the model. A lower AIC value indicates a better-fitting model, balancing model complexity (number of parameters) with goodness of fit.

Changes in gene signature scores (GSS) were calculated as Log2 Fold Change (Log2FC), representing the Log2 of the ratio of surgical score to baseline. Changes in *r* to IS centroids were calculated as the differences between *r* at surgery and baseline. Two-sided t-tests were applied for both paired and unpaired comparisons in POAI and control tumours. p-values less than 0·05 were considered statistically significant. Multiple testing correction was performed using the Benjamini & Hochberg false discovery rate (FDR) method^18^ whenever appropriate. FDR less than 0·05 was considered statistically significant after multiple testing correction. For GSS, a combined significance threshold was defined as P adjusted <0·05 and log2FC (FC)> |0·5| when comparing between the Ki67_2__wk_ High vs Low groups. Assuming a linear relationship between the two variables, Pearson's correlation was used to examine the correlations between changes in gene expression or signature expression levels and changes in Ki67. One-way Analysis of Variance (ANOVA) test was performed to compare GSS between multiple groups. Multivariable ordinal logistic regression was performed on changes in each gene signature expression (GSE) levels, adjusted for key post-surgical clinicopathological covariates as previously mentioned. The models output the odds of having Ki67_2__wk_ High or Low given the changes in GSE levels after 2-week POAI. Chi-square test of independence was performed to test the association between POAI and control groups for the proportion of samples shifting ISs at 2-week.

### Ethics

POETIC trial (ISRCTN: 63882543) was approved by the London–Southeast Research Ethics Committee (reference 08/H1102/37) and carried out according to the Declaration of Helsinki. Patients provided written informed consent to molecular analyses of their samples for research purposes.

### Role of funders

Funders had no role in the study design, data collection, data analyses, interpretation, or report writing.

## Results

### Patient clinicopathological characteristics

A consort diagram is provided in Supplementary Figure S1. Of the 468 patients with ER+/HER2+ tumours, FFPE samples were available for 213 POAI-treated patients and 100 controls. The clinicopathological characteristics were similarly distributed between the two groups (POAI and controls) with FFPE samples (Supplementary Table S2). Histologically, 295 (94·3%) tumours were of the ductal type; 144 (46%) tumours were classified as grade 2 while 156 (49·8%) were grade 3. At the point of surgical intervention, 171 (54·7%) tumours measured between 2 and 5 cm in diameter and 154 (49·2%) exhibited positive nodal status. For adjuvant therapy, 188 (60·1%) patients received both adjuvant chemotherapy and trastuzumab, while 23 (7·3%) patients were treated with chemotherapy without trastuzumab. Associated with advanced age, 98 (31·3%) patients (70·4% in >70 and 29·6% < 70) did not undergo adjuvant trastuzumab or chemotherapy. Almost all patients received adjuvant ET (306, 97·8%). Two patients did not receive any adjuvant treatment, with both having a recurrence event within 50 days from the time of randomisation. In addition, the distribution of patients receiving adjuvant chemotherapy and trastuzumab did not differ based on the Ki67 category (Low vs High; Chi Square test p value = 0·33).

### Impact of changes in Ki67 expression (early AI response) on patient survival outcome

**Fig. 1:**
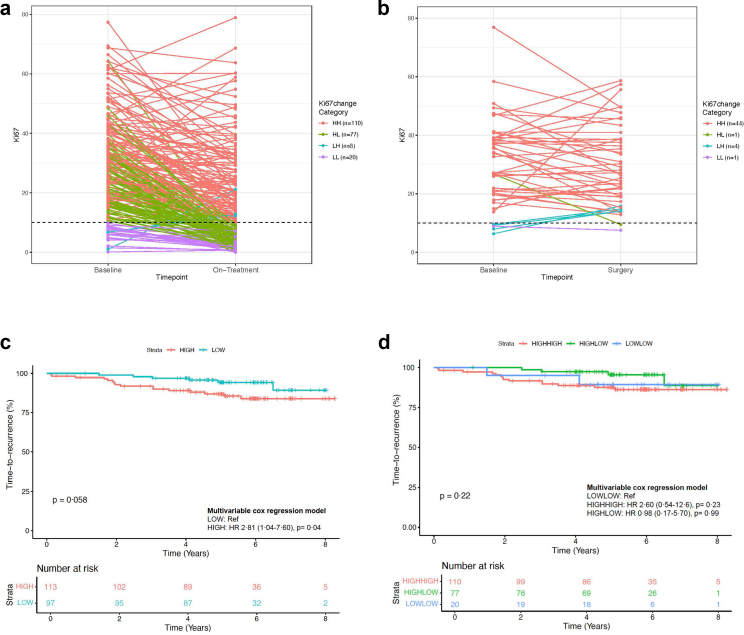
**Ki67 changes**. Line plots of the baseline and surgery/on-treatment Ki67 levels coloured by Ki67 changes categories: High–High (H–H), High-Low (H–L), Low-High (L–H) and Low–Low (L–L) in a. POAI (n = 210) and b. control samples (n = 50). Kaplan Meier curves for TTR in POAI according to c. Ki67_2__wk_ High vs Low and d. Ki67 categories H–H, H-L and L–L. Cox regression analyses were all adjusted by clinicopathological variables.

Paired Ki67 scores were available from 50 Control, selected at random as described in the method section and included in the main clinical study report^7^ as well as for 210 POAI-treated patients. As expected in the control group, 48 (96%) cases had high Ki67_2__wk_ (mean = 28·94%), whereas only 113 (54%) POAI cases demonstrated high Ki67_2__wk_ (mean = 16·47%). Fig. 1a and b depict the classification of Ki67 scores into Low–Low (L–L), Low-High (L-H), High-Low (H-L) and High–High (H–H) distributions. Notably, 42 (88%) cases in the control group were categorised as H–H, and the POAI group primarily fell into H–H (110, 52·4%) and H-L categories (77, 36·7%).

Upon evaluating the association of Ki67 with survival within the POAI group, it was observed that a high (≥10%) vs low (<10%) Ki67_2__wk_ (Fig. 1c), and changes in Ki67 categories (L–L, H-L, H–H) (Fig. 1d) were not associated with differential TTR in Kaplan–Meier analyses (log-rank test). However, Ki67_2__wk_ high (≥10%) suggested an association with worse TTR in univariable cox regression (HR: 2·41; CI (0·94–6·17), p = 0·07). This association was statistically significant in multivariable cox regression after adjustment for clinicopathological variables (HR: 2·81; CI (1·04–7·60), p = 0·04) (Fig. 1c). Furthermore, although baseline Ki67 as a continuous variable showed no association with TTR, on-treatment continuous Ki67_2__wk_ was independently associated with TTR (Table 1).

**Table 1:** Multivariable model comparison including Ki67 at baseline and On-treatment; PAM50 IS at baseline and on-treatment; and Ki67 + PAM50 IS at baseline and on-treatment, respectively in POAI (n = 209).

Variable	Baseline continuous Ki67			On-Treatment continuous Ki67		
	N	HR (95% CI)	p-value	N	HR (95% CI)	p-value
Ki67 (%)	209	1·01 (0·98–1·03)	0·6806	209	1·03 (1·01–1·05)	0·01∗
Age (years)	209	1·07 (1·01–1·12)	0·0131∗	209	1·08 (1·02–1·14)	0·0054∗∗
Tumour size (cm)	209	1·53 (1·22–1·91)	0·0002∗∗∗	209	1·52 (1·21–1·91)	0·0003∗∗∗
Nodal status						
N0 (ref)	111	–	–	111	–	–
N1-3	62	1·11 (0·37–3·37)	0·8546	62	1·52 (0·39–3·59)	0·7563
N4+	36	2·17 (0·83–5·70)	0·1159	36	2·20 (0·84–5·77)	0·1097
Concordance index	0·731 (se = 0·06)			0·765 (se = 0·057)		
Likelihood ratio test	23·82 (p = 0·0002)			29·35 (p = 0·00002)		
AIC	210·731			205·195		

Variable	Baseline PAM50			On-Treatment PAM50		
	N	HR (95% CI)	p-value	N	HR (95% CI)	p-value
PAM50						
LumA (ref)	42	–	–	109	–	–
LumB	75	1·21 (0·30–4·80)	0·7871	14	5·41 (1·50–19·5)	0·0099 ∗∗
HER2-E	89	1·72 (0·47–6·24)	0·4091	83	2·41 (0·87–6·73)	0·0919
Basal	3	4·84 (0·48–49·1)	0·1823	3	6·55 (0·76–56·8)	0·0882
Age (years)	209	1·07 (1·01–1·12)	0·0133∗	209	1·07 (1·02–1·13)	0·01125 ∗
Tumour size (cm)	209	1·50 (1·20–1·88)	0·0004∗∗∗	209	1·53 (1·22–1·92)	0·0003 ∗∗∗
Nodal status						
N0 (ref)	111	–	–	111	–	–
N1-3	62	1·14 (0·37–3·47)	0·8202	62	1·14 (0·38–3·46)	0·8179
N4+	36	2·41 (0·90–6·42)	0·0791	36	2·37 (0·89–6·34)	0·0858
Concordance index	0·742 (se = 0·059)			0·789 (se = 0·053)		
Likelihood ratio test	25·61 (p = 0·0006)			31·43 (p = 0·00005)		
AIC	212·944			207·1192		

Variable	Baseline continuous Ki67 + PAM50			On-Treatment continuous Ki67 + PAM50		
	N	HR (95% CI)	p-value	N	HR (95% CI)	p-value
Ki67 (%)	209	0·99 (0·96–1·03)	0·6546	209	1·02 (0·99–1·05)	0·1883
PAM50						
LumA (ref)	42	–	–	109	–	–
LumB	75	1·35 (0·32–5·76)	0·6875	14	4·11 (1·05–16·0)	0·0418∗
HER2-E	89	2·07 (0·45–9·43)	0·3466	83	1·56 (0·45–5·45)	0·4843
Basal	3	6·68 (0·45–99·2)	0·1676	3	1·93 (0·11–32·5)	0·6477
Age (years)	209	1·06 (1·01–1·12)	0·0181∗	209	1·08 (1·02–1·14)	0·00753∗∗
Tumour size (cm)	209	1·49 (1·19–1·87)	0·00052∗∗∗	209	1·56 (1·23–1·97)	0·00022∗∗∗
Nodal status						
N0 (ref)	111	–	–	111	–	–
N1-3	62	1·12 (0·37–3·44)	0·8422	62	1·17 (0·39–3·52)	0·7805
N4+	36	2·45 (0·91–6·55)	0·0746	36	2·21 (0·82–5·92)	0·1151
Concordance index	0·752 (se = 0·057)			0·789 (se = 0·055)		
Likelihood ratio test	25·81 (p = 0·001)			33·06 (p = 0·00006)		
Changes in Chi-sq against Ki67 only	1·9895 (ns)			3·7045 (ns)		
Changes in Chi-sq against PAM50 only	0·2028 (ns)			1·6287 (ns)		
AIC	214·7412			207·4905		

Abbreviations: AIC, akike information criterion; HR, hazard ratio; HER2-E, HER2 enriched; LumA, Luminal A; LumB, Luminal B. ∗p-value ≤ 0·05, ∗∗p-value ≤ 0·01, ∗∗∗p-value ≤ 0·001.

### Impact of short-duration perioperative aromatase inhibitors on gene expression

**Fig. 2:**
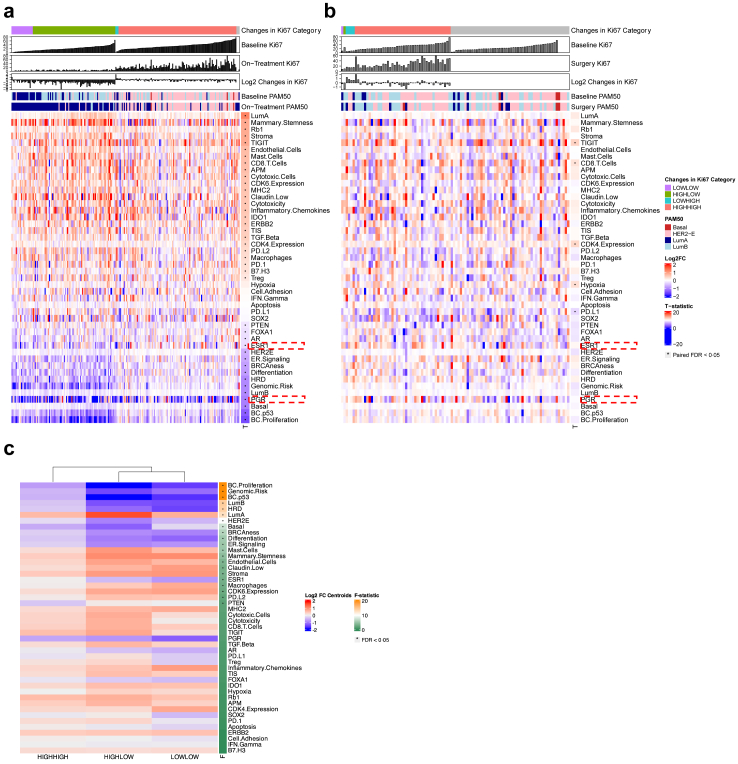
**Gene signature expression changes.** Heatmap of log2 fold change in BC360 biological signature expression with samples sorted by changes in Ki67 categories and baseline Ki67%, for samples in a. POAI (n = 213) and b. control (n = 100) groups. T-tests were performed in control and POAI groups separately for all signatures to compare the expression levels between baseline and surgery/on-treatment. p-values were adjusted by False Discovery Rate (FDR). For heatmap visualisation, Genomic Risk was log2 transformed. Gene signatures were ordered by T-statistic in the POAI group. Higher T-statistic means the signature was upregulated at surgery/on-treatment and lower T-statistic means the signature was upregulated at baseline. c. Hierarchical clustering of BC360 biological signature expression changes from baseline to on-treatment (Log2FC) in POAI (n = 207) supervised by H–H, H-L, L–L Ki67 change categories.

We assessed the GSE profiles at baseline and on-treatment/surgery timepoints to understand the effects of a two-week course of AI treatment on GSE dynamics. There was notable upregulation of tumour immunity signatures, including *TIGIT*, CD8 T-cells, inflammatory chemokines, and *IDO1*, alongside downregulation of proliferation, homologous recombination deficiency (HRD), *TP53* surrogate mutational status signature, and ER-signalling in the POAI group (Fig. 2a). Conversely, samples in the control group exhibited increases in *TIGIT* and CD8 T-cells, along with unique changes such as hypoxia signature upregulation and *PDL1* downregulation (Fig. 2b).

When analysing GSE shifts across Ki67 change categories in the POAI group, there were significant differences, particularly in patients categorised as Ki67 H-L and L–L compared to H–H. In the H-L and L–L categories, there was significant upregulation of tumour immunity and mammary stemness signatures, while markers of proliferation, HRD, *TP53* surrogate mutational status, and ER-signalling were downregulated relative to those in the H–H group (Fig. 2c and Supplementary Figure S2). These GSE alterations were similarly noted when comparing Ki67_2__wk_ High vs Low categories (Supplementary Table S3).

### Impact of short-duration perioperative aromatase inhibitors on intrinsic subtypes

**Fig. 3:**
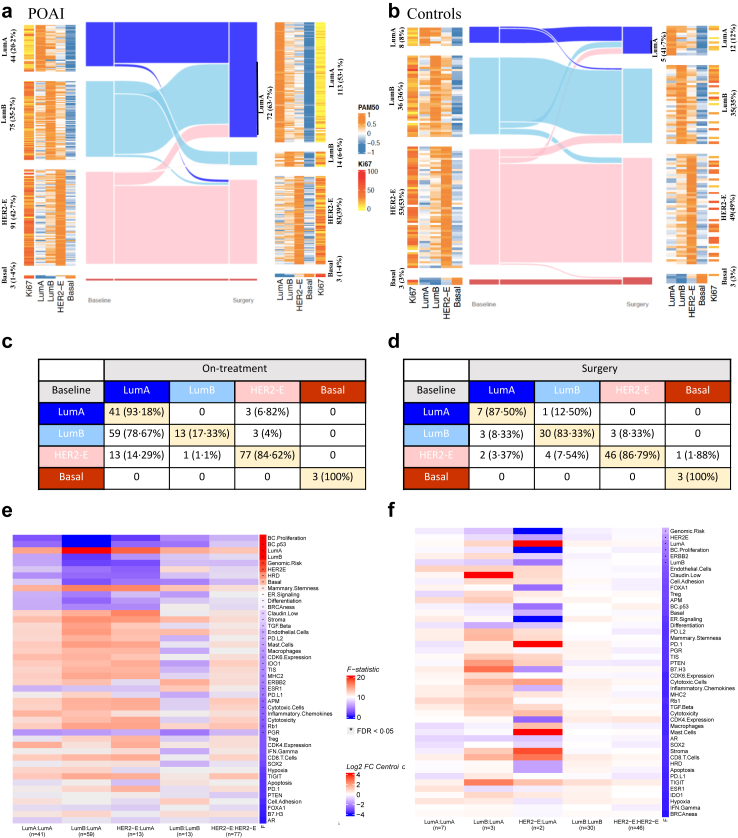
**Intrinsic subtype changes.** Shift of intrinsic subtype from baseline to surgery/on-treatment timepoints in a. POAI (n = 213) and b. control (n = 100) groups. Table showing intrinsic subtype shifts from baseline to on-treatment samples in c. POAI (n = 213) and d. control (n = 100) groups. Supervised hierarchical clustering of changes in gene signature expression shown as mean/standard deviation in POAI (n = 143) (e) and control (n = 88) (f) groups between the 5 Intrinsic subtype change groups. The F-statistic is the ratio of between-group variance and within-group variance. Abbreviations: LumA, Luminal A; LumB, Luminal B; HER2-E, HER2 enriched.

IS shifts were more prevalent from baseline to 2 weeks for the POAI (37%, 79/213) compared to control groups (14%, 14/100; chi-squared test p < 0·001), as expected (Fig. 3). Within the POAI group, 79% of LumB (59/75) and 14% of HER2-E (13/91) were reclassified as LumA during treatment, while the majority of LumA tumours (93%, 41/44) at baseline maintained their classification (Fig. 3a and c). Next, we evaluated whether the reclassifications of ISs in POAI and control were due to borderline correlations with multiple subtypes or true biological change. In POAI, approximately 54% of these tumours shifted towards the IS with the second highest correlation coefficient at baseline (i.e. second most similar categories), with all tumours exhibiting different levels of reduction in proliferation (Fig. 3e, Supplementary Figure S3a). In contrast, the control group showed no significant shifts in IS from baseline to surgery. The ISs shifting in control were predominantly from those with borderline correlation coefficients at baseline, where 11 of the 14 shifts aligned with the second highest baseline correlation coefficient (Fig. 3 and Supplementary Figure S3b).

We then sought to unravel the molecular dynamics induced by short-duration AI treatment, correlating these changes with shifts in ISs (Fig. 3f). In POAI group, tumours transitioning from LumB or HER2-E to LumA exhibited significant molecular alterations, including upregulation of mammary stemness and various immune-related signatures such as *PDL1* or *PDL2*, macrophages, tumour inflammation signature (TIS), *TGF-beta,* and *IDO1*. In contrast, signatures of *TP53* surrogate mutational status, HRD, proliferation and genomic risk were reduced in tumours shifting from LumB or HER2-E at baseline to LumA compared to those that retained their original subtype.

To understand the differential molecular biology in on-treatment LumA based on their baseline characteristics, we compared GSE between on-treatment LumA tumours that were originally LumA and those that had shifted from LumB. As expected, tumours that shifted from LumB to LumA demonstrated higher expression of LumB correlation coefficients, differentiation scores, *ESR1*, genomic risk, and interferon-gamma signatures, and lower expression of LumA score, *PGR*, claudin-low, mast cells, stroma, and mammary stemness compared to tumours that remained LumA throughout treatment (Supplementary Figure S4).

Further analyses revealed a significant distinction between LumB tumours that shifted to LumA and those that retained their original subtype (LumB) (Supplementary Figures S4 and S5a). LumB—LumB tumours had a more aggressive phenotype characterised by higher genomic risk and proliferation scores, correlation coefficients to LumB, HER2-E and basal-like profile, increased expression of *TP53* surrogate mutational status, HRD and differentiation. These tumours also showed lower expression of endothelial cells, stroma, mammary stemness, Lum A and claudin-low signatures compared to those that shifted to LumA (Supplementary Figures S4 and S5a). These observations highlight the critical molecular pathways that underpin IS stability and shifts in response to short-duration ET, offering critical insights for both prognostic assessment and therapeutic targeting in ER+/HER2+ BC.

To evaluate whether the gene signatures were intrinsically different between LumB-LumA and LumB–LumB cases, we compared the baseline GSE between the two groups. Cases that remained LumB had higher *TP53* surrogate mutation status score (FDR = 0·04) and lower expression of *PTEN* (FDR = 0·04).

In the control group, only minimal significant GSE changes were observed across IS transitioning categories. These observed differences were primarily driven by the downregulation of genomic risk score and *ERBB2 e*xpression in the two cases that transitioned from HER2-E to LumA, likely reflecting variability introduced by different sampling methods (i.e., biopsy vs surgery) and the small number of cases (Fig. 3f and Supplementary Figure S5b).

### Impact of gene signature expression changes on risk of recurrence

We evaluated the impact of on-treatment GSE levels on clinical outcomes within the POAI cohort. Only *TGF-beta* expression was significantly associated with TTR (Supplementary Table S4). Further analyses of changes in GSE with TTR suggested no significant associations of GSE with TTR (Supplementary Figure S6).

Next, we evaluated the changes in GSE, their relationship with changes in Ki67 (IHC expression as a continuous variable) and association with TTR. As expected, changes in signatures that positively correlated with Ki67 exhibited similar hazard ratios (HR) to the HR observed in Ki67 Cox Regression model (HR = 1·64) adjusted for clinicopathological parameters. Conversely, signatures negatively correlated with Ki67 displayed corresponding HR that were less than 1 (Supplementary Figure S6a). Only four signatures reached statistical significance in Cox regression models before multiple testing correction, but none of them were significant after FDR adjustment. In addition, incorporating GSE changes did not provide additional prognostic information to a multi-variable model with Ki67. However, including Ki67 changes in the multi-variable Cox model with GSE models can provide additional prognostic information (Supplementary Figure S6b).

### Association of molecular subtype with risk of recurrence

**Fig. 4:**
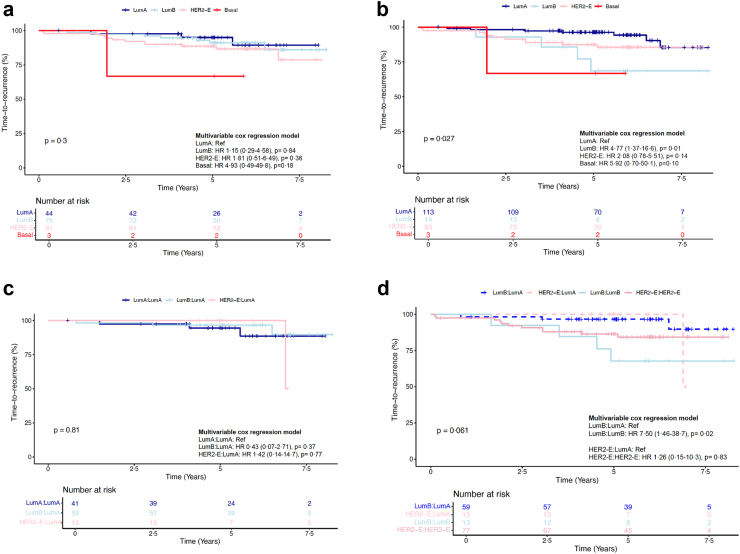
**Intrinsic subtype and time to recurrence in POAI.** Kaplan Meier curves for TTR according to intrinsic subtypes (4 classes) at a. baseline (n = 213), b. on-treatment timepoints (LumA as reference) (n = 213). c. Kaplan Meier curves for TTR according to LumA–LumA vs LumB-LumA (Univariable and multivariable cox regression analysis adjusted by clinicopathological variables, LumA–LumA as reference) (n = 113). d. Kaplan Meier curves for TTR according to LumB:LumA vs LumB:LumB and HER2-E:LumA vs HER2-E:HER2-E (LumB:LumA and HER2-E:LumA as reference) (n = 162). Cox regression analyses were all adjusted by clinicopathological variables.

To determine the clinical significance of the on-treatment IS, we assessed its association with TTR. As previously reported, in the POAI group,^13^ there was no association observed between the four ISs at baseline with TTR (Fig. 4a). However, the on-treatment LumA IS was associated with more favourable TTR compared to LumB (HR 0·2; CI 95% 0·06–0·72, p = 0·01) (Fig. 4b and d). For patients with an on-treatment LumA subtype, their TTR did not differ based on whether their baseline subtype was HER2-E, LumB or LumA (Fig. 4c).

After adjusting for standard clinicopathological variables, including age, post-treatment tumour size, and nodal status, the on-treatment LumA IS remained as an independent predictor of better TTR compared to LumB and HER2-E (Table 1).

To assess the potential enhancement of prognostic precision, we applied a Cox regression model integrating both Ki67 and ISs at both Baseline and 2wk in the POAI group. This combined model did not provide additional prognostic information (Table 1). These results suggested each biomarker independently captured similar prognostic data, while a limited incremental benefit was shown from their combined use in predicting outcomes within this cohort.

## Discussion

Established research, including findings from Dowsett *et al*.,^19^ highlights that ER+ BCs overexpressing HER2 often show limited responsiveness to ET, placing these patients at increased risk of relapse, particularly when they do not achieve a complete response to anti-HER2 therapies.^20^ While much of the existing research on ER+/HER2+ BC has concentrated on resistance to anti-HER2 therapies, the mechanisms behind resistance to AI in this subgroup remain less explored. Our study delivers a detailed examination of inter-tumour heterogeneity, specifically analysing the response of ISs to short duration of AI and the subsequent shifts in molecular profile relative to long-term clinical outcomes.

Data from the POETIC trial showed that while high baseline Ki67 levels in ER+/HER2− tumours that dropped post POAI correlated with lower risk of recurrence measured by Ki67 categories, this was not seen in the ER+/HER2+ group, maybe because the anti-HER2 therapy they received compensated for or overcame the ET resistance. This difference emphasises the need for AI resistance research to uncover predictive biomarkers and improve treatments in ER+/HER2+ BC.

Our study shows a moderate correlation between dichotomised and continuous Ki67_2__wk_ and risk of recurrence in this ER+/HER2+ subset of POETIC trial patients and elucidates the prognostic significance of on-treatment Ki67. The borderline association of dichotomised Ki67_2__wk_ and the difficulty of implementing continuous variables as a tool in clinical practice underscores the importance to further investigate molecular markers as both predictive and prognostic tools in ER+/HER2+ BC.

Our previous molecular analyses within the POETIC trial have identified the baseline HER2-E subtype as a statistically significant predictor of resistance to aromatase inhibitors in ER+/HER2+ BC that is also associated with an increased risk of relapse relative to Luminal tumours. However, this relationship between baseline HER2-E and poorer prognosis seemed to be mitigated upon controlling for the types of adjuvant treatments administered.^13^

Our research builds on previous studies investigating shifts in IS post-treatment, which typically ranged from 2 weeks to several months, and involved heterogenous treatment regimens, including different combinations of anti-HER2 therapies with or without AIs.^21^^,^^22^

Prior trials, such as the NBRST trial, enrolled 297 HER2+ patients who received neoadjuvant chemotherapy combined with HER2-targeted therapy and subsequently underwent surgery. In this trial, the highest pCR rate was observed in BluePrint HER2-type patients treated with trastuzumab plus pertuzumab (76%).^23^ A related finding was published in 2021 as part of the ADAPT HER2+ trial in which the pooled TDM1 arm of patients with HER2-E subtype had a higher pCR, which was not observed in the trastuzumab arm.^24^

Unlike these prior studies, our study focused on the impact of a 2-week AI-only regimen. We found that most LumA tumours maintained their subtype with a reduction in proliferation scores, suggesting that reassessing LumA at surgery post-short-term AI is unlikely to influence clinical decision-making due to their already favourable outcomes. In this line, a single-arm, multicentre study (PerELISA) included 65 postmenopausal women with operable HR+/HER2+ breast cancer, who received two weeks of letrozole followed by re-biopsy for Ki67 evaluation. This study reported a strong association between PAM50 intrinsic subtyping and molecular responders (defined as a Ki67 relative reduction of >20% from baseline). Notably, 92% of responders were classified as luminal A or B, compared to only 44% of HER2-E and basal-like subtypes (p < 0·001), however, no data on survival was reported.^25^ Therefore, our results corroborate previous findings and suggest that the Luminal A subtype of ER+/HER2+ breast cancer may benefit from AI plus anti-HER2 therapy alone, potentially achieving long-term survival without the need for chemotherapy.

In contrast, the majority of LumB (78%) tumours shifted to LumA, mirroring the high transition rate seen in patients with ER+/HER2−breast cancer in the POETIC & NeoAI cohort (>1-month neoadjuvant NT),^26^ indicating similar AI responsiveness regarding these ISs irrespective of HER2 status. However, the transition was significantly less common in HER2-E tumours of the ER+/HER2+ subset, where only 14% shifted to LumA after AI treatment, compared to a 50% transition in the POETIC ER+/HER2− group. This suggests a possible greater resistance to AI in HER2-E tumours and reflects molecular differences of this IS according to HER2 status.

In addition, the IS correlation coefficient score showed that AI-sensitive tumours, defined by reduction of Ki67, typically characterised gene expression profiles closer to LumA. Control cases that shifted their IS, often had borderline correlation coefficients at baseline or during treatment, emphasizing the role of intra-tumoral and inter-tumoral heterogeneity on leading to differences even in clinical grade molecular profiling in a small proportion of cases. Indeed, only minimal significant GSE changes were observed across IS transitioning categories in controls.^27^

The prognostic value of the IS post-2 weeks of AI treatment, with tumour shifted to LumA on-treatment associated with lower risk of relapse, underscores the clinical relevance and possible utility of assessing IS at the on-treatment timepoint in those non-LumA tumours at diagnosis. While on-treatment LumA IS consistently predicts better TTR irrespective of baseline IS, our findings indicate biological distinctions among on-treatment LumA tumours. Tumours initially classified as LumB that transition to LumA on-treatment were characterised with slightly more aggressive biology compared to those that were LumA at both timepoints. Conversely, LumB tumours that do not transition to LumA display significantly more aggressive characteristics.

These findings support the utility of genomic testing in refining risk stratification and informing treatment decisions in ER+/HER2+ breast cancer. Established assays such as Prosigna, Mammaprint, Oncotype DX, and EndoPredict provide valuable insights into tumour biology, recurrence risk, and likely response to endocrine or chemotherapy in ER+/HER2− early-stage breast cancer. However, their role in HER2+ disease remains unclear. Similarly, while HER2DX^28^ offers prognostic insights specific to HER2+ disease, it does not provide information on response to AI therapy in this setting. Our study highlights an important perspective, emphasizing the clinical relevance of dynamic changes in IS during treatment. By incorporating treatment-induced biological shifts, this approach complements existing genomic tests and has the potential to guide more precise therapeutic strategies, particularly in ER+/HER2+ disease, where the application of genomic tools remains underexplored.

Prior studies of ER+/HER2− disease suggest that AI has an immune-modulating effect, leading to an enrichment of tumour-infiltrating cells and immune-related characteristics, as well as emphasizing the importance of tumour microenvironment in cancer progression and therapeutic responses.^29^, ^30^, ^31^, ^32^, ^33^ Our study extends these observations to ER+/HER2+ disease. We demonstrate similar immune activation in ER+/HER2+ tumours, including upregulation of immune-checkpoint inhibitors related signatures such as *PD1*, *PDL2,* *TIGIT* or *IDO1* and other immune-related genes such as *TGF-beta* or macrophages particularly in tumours showing higher sensitivity to AI as indicated by Ki67 reductions. Noteworthy, minimal significant GSE changes were also observed in the control group. *TP53* mutations are recognised as biomarkers linked with resistance to ET.^34^ Our study shows a more significant downregulation of the *TP53* surrogate mutational status signature in patients exhibiting a reduction in Ki67. As anticipated, endocrine-related signalling pathways were also diminished, with a particularly notable decrease in *PGR*.

So far as we are aware, this is the largest molecular study of endocrine resistance/sensitivity in ER+/HER2+ tumours. Our study provides many insight*s* but has some limitations. While our analyses were limited to the BC360 platform, potentially missing some genes, this focused approach allowed us to precisely evaluate molecular changes under AI treatment and perform robust comparisons across the entire cohort of ER+/HER2+ in the POETIC trial. Although the number of TTR events in our cohort was relatively small, reflecting the generally favourable prognosis of the studied population, this does not undermine the significance of our findings. Even with a limited number of events, we were able to detect meaningful prognostic differences. Specifically, we observed a significantly better prognosis for LumA tumours compared to other subtypes, reinforcing the robustness and validity of our results. These findings remain relevant despite the lower number of events, highlighting the strength of the observed associations. However, there may be other more modest but clinically meaningful effects that are not detected due to the small number of events.

Lastly, we acknowledge that the POETIC trial was conducted before the incorporation of dual HER2 blockade (trastuzumab plus pertuzumab) into standard neoadjuvant therapy for HER2-positive early breast cancer or the adjuvant TDM1 in residual disease.^35^ However, the biological features evaluated in our analysis—including early endocrine response and gene expression patterns—remain mechanistically relevant regardless of the specific anti-HER2 regimen used. The HER2-positive patients in POETIC had early-stage disease and received the standard of care available at the time, which included trastuzumab in most cases. Despite differences in systemic therapy over time, the diagnostic methodologies applied are consistent with current clinical practice. Importantly, our findings offer valuable insights into the biology of HER2-positive tumours within the endocrine window-of-opportunity setting—a context that remains underexplored. These insights are highly relevant to contemporary treatment strategies, particularly in light of emerging evidence supporting the addition of CDK4/6 inhibitors to address hormone resistance in ER+/HER2+ breast cancer. Incorporating these inhibitors to enhance the efficacy of endocrine therapy in early-stage AI-resistant tumours, specifically on-treatment LumB, may represent a promising treatment strategy. Conversely, the potentially poorer outcomes observed in on-treatment HER2-E patients may already be addressed through the current use of neoadjuvant anti-HER2 double blockade therapies, such as pertuzumab in combination with trastuzumab or T-DM1, in cases of residual disease. Early identification of such resistance is crucial, and the POETIC trial uniquely facilitates this by identifying patients who do not respond to early AI therapy, with all participants having received at least five years of endocrine therapy. These advancements may impact the generalizability of our findings regarding long-term outcomes for clinicopathological variables defining high-risk ER+/HER2+ tumour. Our findings provide valuable insights into de-escalation treatment strategies, particularly for Luminal A tumours, such as reducing or avoiding chemotherapy use. Notably, a subset of the population in our study did not receive anti-HER2 therapy, yet Luminal A tumours still exhibited a better prognosis. This observation reinforces the favourable biology of the IS and underscores their potential to achieve excellent outcomes even in the absence of intensive systemic treatments, further supporting tailored and less aggressive therapeutic approaches in this subgroup that could help to reduce over-treatment in HER2+ disease.

The large size of this study provides the opportunity for new insights into global gene expression changes that closely reflect clinical practices, enhancing the translational value of our findings. Additionally, including a control group in our research design allows us to distinguish the genuine effects of AI therapy from artefactual changes related to sample taking and processing. Our study delves into the molecular responses to short-duration AI treatments in this specific subset of BC, offering promising directions for tailored treatment approaches and a deeper understanding of the underlying tumour biology in response to ET. This positions our study as a valuable resource for advancing treatment strategies and optimizing patient outcomes in BC care.

### Conclusions

Our study provides a comprehensive picture of the dynamic changes in early ER+/HER2+ BC in response to ET, highlighting the clinical impact of perioperative AI in this patient subset.

Our findings suggest that assessing ISs just 2 weeks after starting AI treatment can be a crucial step in identifying patients with a lower risk of relapse. Maintaining and/or transitioning to a LumA subtype at surgery underscores its potential to guide the selection of patients for tailored therapeutic strategies. Specifically, these insights could inform decisions to de-escalate treatment for patients demonstrating favourable prognostic indicators or, conversely, to escalate interventions for those persisting with an on-treatment LumB subtype, or anti-HER2 treatment with an on-treatment HER2-E subtype. This approach promises to refine personalised treatment plans, enhancing outcomes and optimizing therapeutic efficacy in clinical practice.

## Contributors

MB and ELK designed, conducted part of the research work, verified the data and wrote the original draft of the manuscript. XZ analysed and verified the data and wrote the original draft of the manuscript. HT and LK participated in study design, data collection and interpretation. CH, assisted with participant recruitment and data collection and was a Trial Management Group member. JMB has provided oversight and guidance for trial management, statistics and data interpretation throughout the trial including insights into the translational research project presented here. AA conducted part of the research work. IS participated in the design of the original POETIC trial and was chief investigator of it. ES supervised some of the analysis of the data. MD was involved in the investigation and visualisation of the study. MCUC designed and led the study. She also led the conceptualisation, methodology, resources and funding acquisition, data curation and statistical analysis plan, supervision. All authors made substantial contributions to the manuscript, read and approved the final version of the manuscript.

## Data sharing statement

De-identified data will be made available to other researchers on request, subject to approval of a formal data access request in accordance with the ICR-CTSU data and sample access policy. Trial documentation including the protocol and gene expression data are available on request by contacting poetic-icrctsu@icr.ac.uk. The ICR-CTSU supports the wider dissemination of information from the research it does, and increased cooperation between investigators. Trial data is collected, managed, stored, shared, and archived according to ICR-CTSU Standard Operating Procedures in order to ensure the enduring quality, integrity, and utility of the data. Formal requests for data sharing are considered in line with the Institute of Cancer Research Clinical Trials and Statistics Unit (ICR-CTSU) procedures with due regard given to funder and sponsor guidelines. Requests are via a standard proforma describing the nature of the proposed research and extent of data requirements. Data recipients are required to enter a formal data sharing agreement which describes the conditions for release and requirements for data transfer, storage, archiving, publication and intellectual property. Requests are reviewed by the Trial Management Group (TMG) in terms of scientific merit and ethical considerations including patient consent. Data sharing is allowed if proposed projects have a sound scientific or patient benefit rationale as agreed by the TMG and approved by the Trial Steering Committee as required. Restrictions relating to patient confidentiality and consent will be limited by aggregating and anonymising identifiable patient data. Additionally, all indirect identifiers that might lead to deductive disclosures will be removed in line with Cancer Research UK Data Sharing Guidelines. Additional documents might be shared if approved by the TMG and Trial Steering Committee (e.g., statistical analysis plan and informed consent form).

## Declaration of interests

M.B.S declares funding from Fundación Alfonso Martin Escudero, Rio Hortega contracts 2022/2023 CM22/00101 and the 2024 BBVA Foundation/Hospital Clinic of Barcelona Joan Rodés-Josep Baselga Advanced Research Contracts in Oncology for Milana Bergamino's fellowship funding. She also declares personal funding from Pfizer, Novartis and Astra Zeneca and travel expenses from Novartis, Lilly and Astra Zeneca for educational events and advisories. M.C.U.C. is an inventor on US Patent No. 9,631,239 (“Method of classifying a breast cancer intrinsic subtype”) with royalties paid. She is also an inventor on a pending patent application (PCT/EP2021/07368) related to a CDK4/6 inhibitor sensitivity assay. Additionally, she serves in an advisory role for Veracyte. H.T. declares receiving salary support from AstraZeneca UK Ltd, Eli Lilly & Co Limited, Pfizer Inc., Aventis Pharma Limited (Sanofi), the National Institute for Health Research, the Higher Education Funding Council, Cancer Research UK, and Prostate Cancer UK. JMB reports grants from Cancer Research UK, during the conduct of the study; grants from Medivation; grants and non-financial support from AstraZeneca, Merck Sharp & Dohme, Puma Biotechnology, Clovis Oncology, Pfizer, Janssen-Cilag, Novartis, Lilly and Roche, outside the submitted work. LK reports grant from Cancer Research UK and the National Institute for Health and Care Research, during the conduct of the study. CH declares payment for expert testimony from the General Medical Council and honoraria from Exact Science. EFS declares a pending patent application (PCT/EP2021/07368) related to a CDK4/6 inhibitor sensitivity assay. M.D. receives compensation through the ICR Rewards for Inventors Scheme for abiraterone and is an inventor on a pending patent application (PCT/EP2021/07368) related to a CDK4/6 inhibitor sensitivity assay. The other authors declare no competing interests.

## Appendix A Supplementary data

Supplementary Figures and Tables

POETIC Protocol Version6
