# Supplementary material for: Short-duration preoperative endocrine therapy alters molecular profiles to predict favourable outcome in ER+/HER2+ early breast cancer: a POETIC translational study
Source: eBioMedicine. 2025 Jul 18;118:105823. doi: 10.1016/j.ebiom.2025.105823 (PMC12368290; doi:10.1016/j.ebiom.2025.105823)
Supplement: Supplementary Figures and Tables [file mmc1.pptx]

## Slide 1
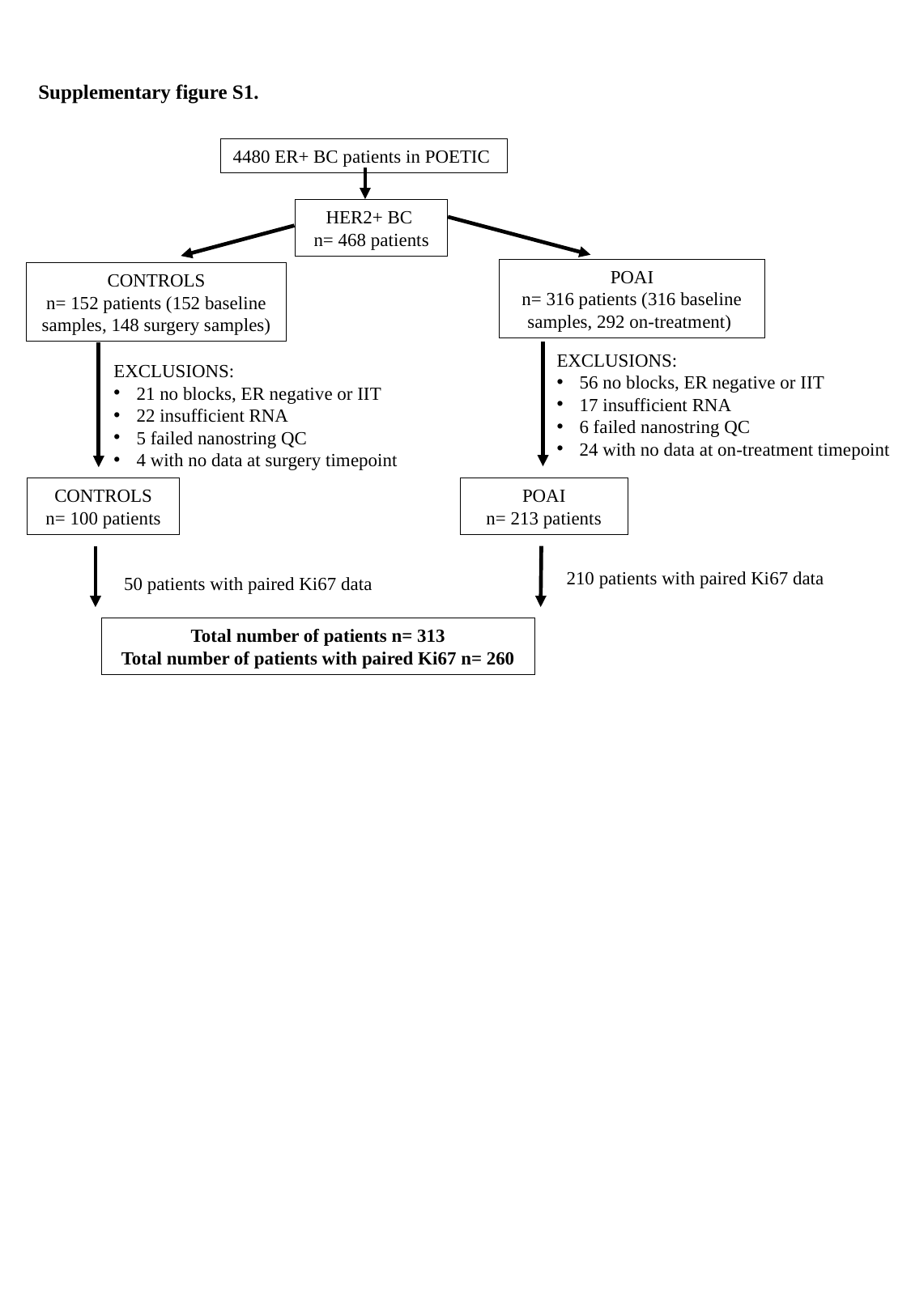

Supplementary figure S1.
4480 ER+ BC patients in POETIC
HER2+ BC
n= 468 patients
POAI
n= 316 patients (316 baseline samples, 292 on-treatment)
CONTROLS
n= 152 patients (152 baseline samples, 148 surgery samples)
EXCLUSIONS:
56 no blocks, ER negative or IIT
17 insufficient RNA
6 failed nanostring QC
24 with no data at on-treatment timepoint
EXCLUSIONS:
21 no blocks, ER negative or IIT
22 insufficient RNA
5 failed nanostring QC
4 with no data at surgery timepoint
CONTROLS
n= 100 patients
POAI
n= 213 patients
210 patients with paired Ki67 data
50 patients with paired Ki67 data
Total number of patients n= 313
Total number of patients with paired Ki67 n= 260

## Slide 2
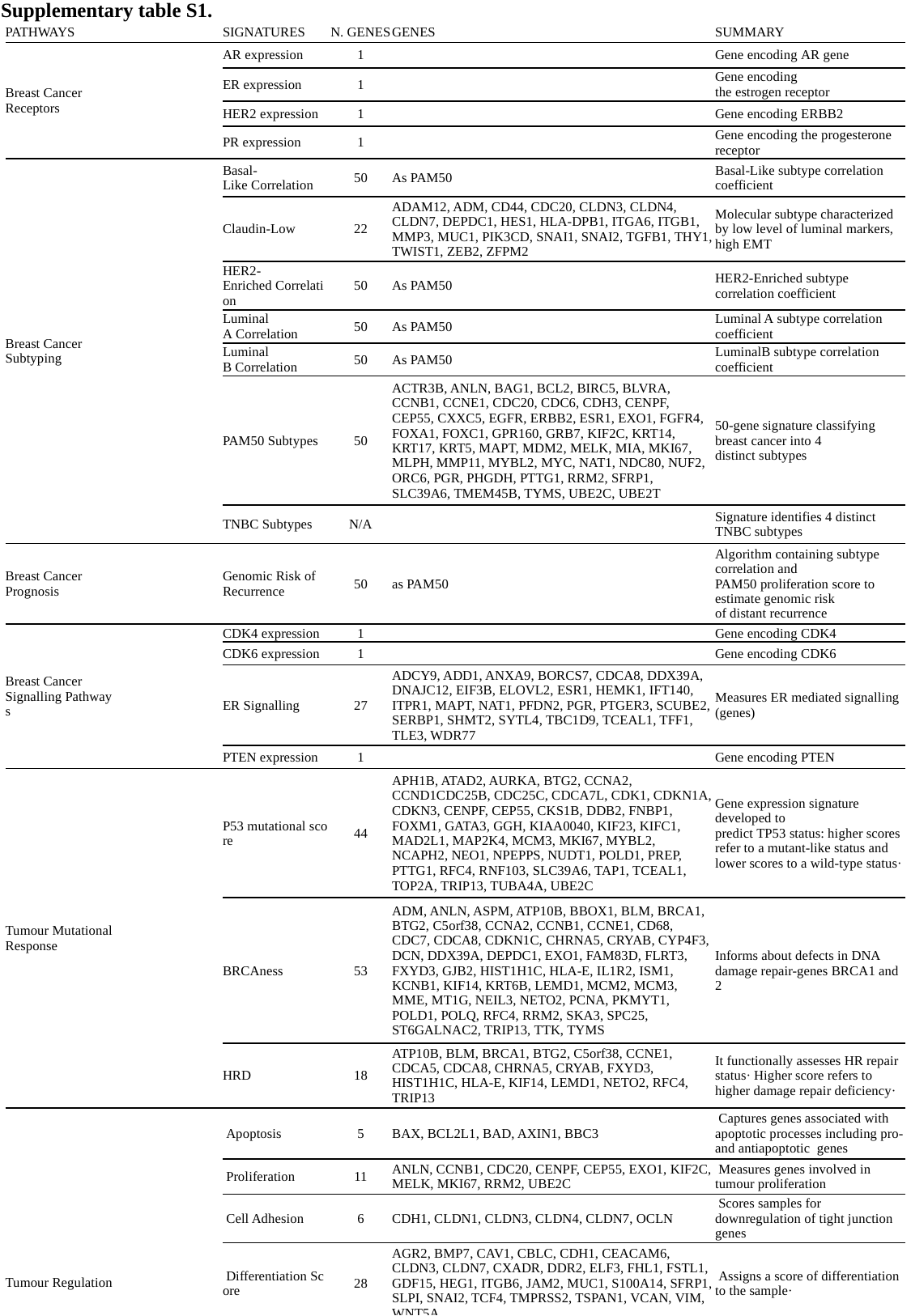

Supplementary table S1.
| PATHWAYS | | SIGNATURES | N. GENES | GENES | SUMMARY |
| --- | --- | --- | --- | --- | --- |
| Breast Cancer Receptors | | AR expression | 1 | | Gene encoding AR gene |
| | | ER expression | 1 | | Gene encoding the estrogen receptor |
| | | HER2 expression | 1 | | Gene encoding ERBB2 |
| | | PR expression | 1 | | Gene encoding the progesterone receptor |
| Breast Cancer Subtyping | | Basal-Like Correlation | 50 | As PAM50 | Basal-Like subtype correlation coefficient |
| | | Claudin-Low | 22 | ADAM12, ADM, CD44, CDC20, CLDN3, CLDN4, CLDN7, DEPDC1, HES1, HLA-DPB1, ITGA6, ITGB1, MMP3, MUC1, PIK3CD, SNAI1, SNAI2, TGFB1, THY1, TWIST1, ZEB2, ZFPM2 | Molecular subtype characterized by low level of luminal markers, high EMT |
| | | HER2-Enriched Correlation | 50 | As PAM50 | HER2-Enriched subtype correlation coefficient |
| | | Luminal A Correlation | 50 | As PAM50 | Luminal A subtype correlation coefficient |
| | | Luminal B Correlation | 50 | As PAM50 | LuminalB subtype correlation coefficient |
| | | PAM50 Subtypes | 50 | ACTR3B, ANLN, BAG1, BCL2, BIRC5, BLVRA, CCNB1, CCNE1, CDC20, CDC6, CDH3, CENPF, CEP55, CXXC5, EGFR, ERBB2, ESR1, EXO1, FGFR4, FOXA1, FOXC1, GPR160, GRB7, KIF2C, KRT14, KRT17, KRT5, MAPT, MDM2, MELK, MIA, MKI67, MLPH, MMP11, MYBL2, MYC, NAT1, NDC80, NUF2, ORC6, PGR, PHGDH, PTTG1, RRM2, SFRP1, SLC39A6, TMEM45B, TYMS, UBE2C, UBE2T | 50-gene signature classifying breast cancer into 4 distinct subtypes |
| | | TNBC Subtypes | N/A | | Signature identifies 4 distinct TNBC subtypes |
| Breast Cancer Prognosis | | Genomic Risk of Recurrence | 50 | as PAM50 | Algorithm containing subtype correlation and PAM50 proliferation score to estimate genomic risk of distant recurrence |
| Breast Cancer  Signalling Pathways | | CDK4 expression | 1 | | Gene encoding CDK4 |
| | | CDK6 expression | 1 | | Gene encoding CDK6 |
| | | ER Signalling | 27 | ADCY9, ADD1, ANXA9, BORCS7, CDCA8, DDX39A, DNAJC12, EIF3B, ELOVL2, ESR1, HEMK1, IFT140, ITPR1, MAPT, NAT1, PFDN2, PGR, PTGER3, SCUBE2, SERBP1, SHMT2, SYTL4, TBC1D9, TCEAL1, TFF1, TLE3, WDR77 | Measures ER mediated signalling (genes) |
| | | PTEN expression | 1 | | Gene encoding PTEN |
| Tumour Mutational Response | | P53 mutational score | 44 | APH1B, ATAD2, AURKA, BTG2, CCNA2, CCND1CDC25B, CDC25C, CDCA7L, CDK1, CDKN1A, CDKN3, CENPF, CEP55, CKS1B, DDB2, FNBP1, FOXM1, GATA3, GGH, KIAA0040, KIF23, KIFC1, MAD2L1, MAP2K4, MCM3, MKI67, MYBL2, NCAPH2, NEO1, NPEPPS, NUDT1, POLD1, PREP, PTTG1, RFC4, RNF103, SLC39A6, TAP1, TCEAL1, TOP2A, TRIP13, TUBA4A, UBE2C | Gene expression signature developed to predict TP53 status: higher scores refer to a mutant-like status and lower scores to a wild-type status· |
| | | BRCAness | 53 | ADM, ANLN, ASPM, ATP10B, BBOX1, BLM, BRCA1, BTG2, C5orf38, CCNA2, CCNB1, CCNE1, CD68, CDC7, CDCA8, CDKN1C, CHRNA5, CRYAB, CYP4F3, DCN, DDX39A, DEPDC1, EXO1, FAM83D, FLRT3, FXYD3, GJB2, HIST1H1C, HLA-E, IL1R2, ISM1, KCNB1, KIF14, KRT6B, LEMD1, MCM2, MCM3, MME, MT1G, NEIL3, NETO2, PCNA, PKMYT1, POLD1, POLQ, RFC4, RRM2, SKA3, SPC25, ST6GALNAC2, TRIP13, TTK, TYMS | Informs about defects in DNA damage repair-genes BRCA1 and 2 |
| | | HRD | 18 | ATP10B, BLM, BRCA1, BTG2, C5orf38, CCNE1, CDCA5, CDCA8, CHRNA5, CRYAB, FXYD3, HIST1H1C, HLA-E, KIF14, LEMD1, NETO2, RFC4, TRIP13 | It functionally assesses HR repair status· Higher score refers to higher damage repair deficiency· |
| Tumour Regulation | | Apoptosis | 5 | BAX, BCL2L1, BAD, AXIN1, BBC3 | Captures genes associated with apoptotic processes including pro- and antiapoptotic  genes |
| | | Proliferation | 11 | ANLN, CCNB1, CDC20, CENPF, CEP55, EXO1, KIF2C, MELK, MKI67, RRM2, UBE2C | Measures genes involved in tumour proliferation |
| | | Cell Adhesion | 6 | CDH1, CLDN1, CLDN3, CLDN4, CLDN7, OCLN | Scores samples for downregulation of tight junction genes |
| | | Differentiation Score | 28 | AGR2, BMP7, CAV1, CBLC, CDH1, CEACAM6, CLDN3, CLDN7, CXADR, DDR2, ELF3, FHL1, FSTL1, GDF15, HEG1, ITGB6, JAM2, MUC1, S100A14, SFRP1, SLPI, SNAI2, TCF4, TMPRSS2, TSPAN1, VCAN, VIM, WNT5A | Assigns a score of differentiation to the sample· |
| | | FOXA1 expression | 1 | | Gene encoding FOXA1 transcription factor |
| | | Mammary Stemness | 10 | CAV1, DDR2, FHL1, FSTL1, HEG1, JAM2, SNAI2, TCF4, VCAN, VIM | Measures a cluster of EMT genes upregulated in stem-cell-like tumours |
| | | Rb1 Expression | 1 | | Gene encoding retinoblastoma gene |
| | | SOX2 Expression | 1 | | Gene encoding SRY-box2 |

## Slide 3
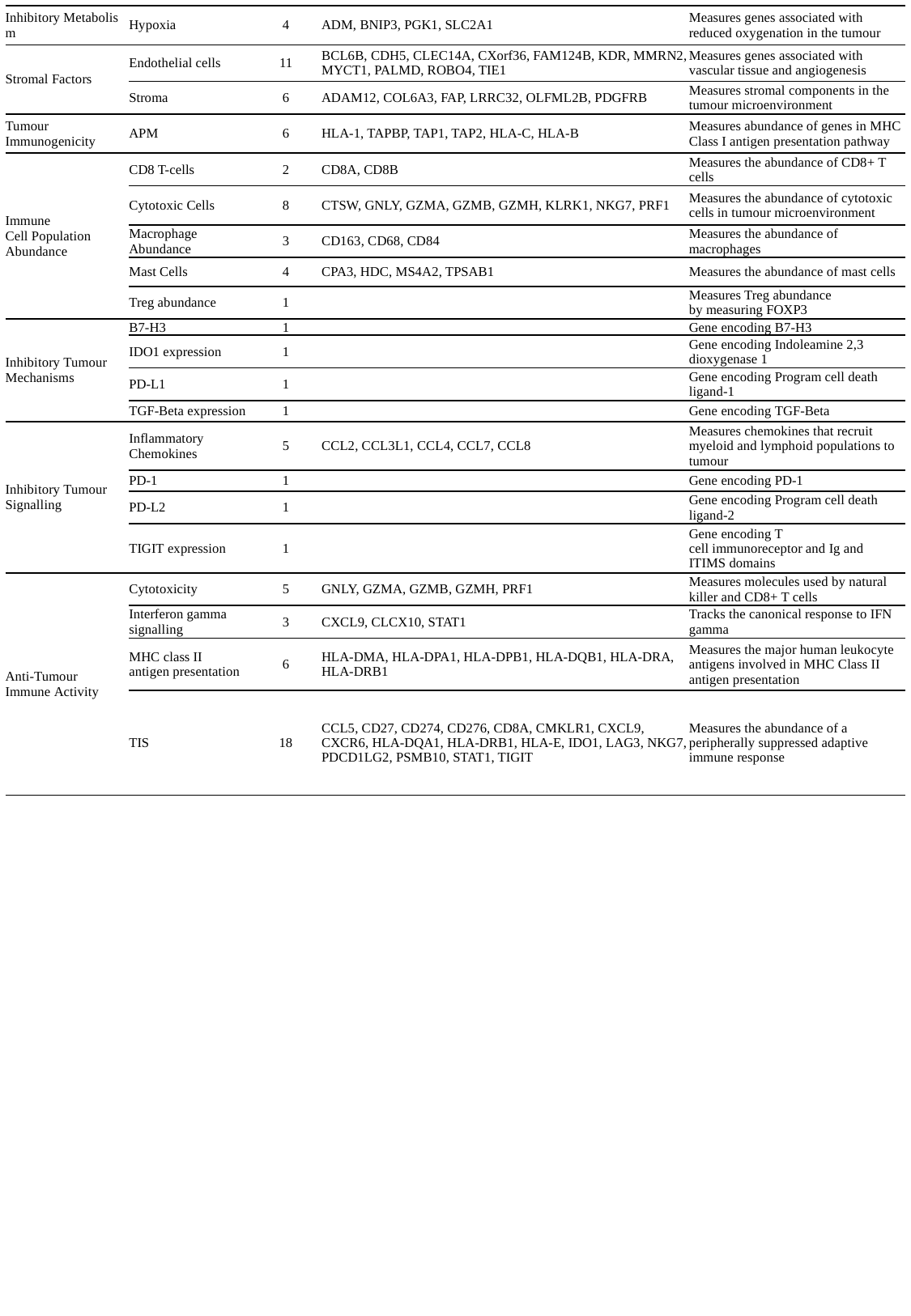

| Inhibitory Metabolism | Hypoxia | 4 | ADM, BNIP3, PGK1, SLC2A1 | Measures genes associated with reduced oxygenation in the tumour |
| --- | --- | --- | --- | --- |
| Stromal Factors | Endothelial cells | 11 | BCL6B, CDH5, CLEC14A, CXorf36, FAM124B, KDR, MMRN2, MYCT1, PALMD, ROBO4, TIE1 | Measures genes associated with vascular tissue and angiogenesis |
| | Stroma | 6 | ADAM12, COL6A3, FAP, LRRC32, OLFML2B, PDGFRB | Measures stromal components in the tumour microenvironment |
| Tumour Immunogenicity | APM | 6 | HLA-1, TAPBP, TAP1, TAP2, HLA-C, HLA-B | Measures abundance of genes in MHC Class I antigen presentation pathway |
| Immune Cell Population  Abundance | CD8 T-cells | 2 | CD8A, CD8B | Measures the abundance of CD8+ T cells |
| | Cytotoxic Cells | 8 | CTSW, GNLY, GZMA, GZMB, GZMH, KLRK1, NKG7, PRF1 | Measures the abundance of cytotoxic cells in tumour microenvironment |
| | Macrophage  Abundance | 3 | CD163, CD68, CD84 | Measures the abundance of macrophages |
| | Mast Cells | 4 | CPA3, HDC, MS4A2, TPSAB1 | Measures the abundance of mast cells |
| | Treg abundance | 1 | | Measures Treg abundance by measuring FOXP3 |
| Inhibitory Tumour  Mechanisms | B7-H3 | 1 | | Gene encoding B7-H3 |
| | IDO1 expression | 1 | | Gene encoding Indoleamine 2,3 dioxygenase 1 |
| | PD-L1 | 1 | | Gene encoding Program cell death ligand-1 |
| | TGF-Beta expression | 1 | | Gene encoding TGF-Beta |
| Inhibitory Tumour  Signalling | Inflammatory  Chemokines | 5 | CCL2, CCL3L1, CCL4, CCL7, CCL8 | Measures chemokines that recruit myeloid and lymphoid populations to tumour |
| | PD-1 | 1 | | Gene encoding PD-1 |
| | PD-L2 | 1 | | Gene encoding Program cell death ligand-2 |
| | TIGIT expression | 1 | | Gene encoding T cell immunoreceptor and Ig and ITIMS domains |
| Anti-Tumour  Immune Activity | Cytotoxicity | 5 | GNLY, GZMA, GZMB, GZMH, PRF1 | Measures molecules used by natural killer and CD8+ T cells |
| | Interferon gamma  signalling | 3 | CXCL9, CLCX10, STAT1 | Tracks the canonical response to IFN gamma |
| | MHC class II  antigen presentation | 6 | HLA-DMA, HLA-DPA1, HLA-DPB1, HLA-DQB1, HLA-DRA, HLA-DRB1 | Measures the major human leukocyte antigens involved in MHC Class II antigen presentation |
| | TIS | 18 | CCL5, CD27, CD274, CD276, CD8A, CMKLR1, CXCL9, CXCR6, HLA-DQA1, HLA-DRB1, HLA-E, IDO1, LAG3, NKG7, PDCD1LG2, PSMB10, STAT1, TIGIT | Measures the abundance of a peripherally suppressed adaptive immune response |

## Slide 4
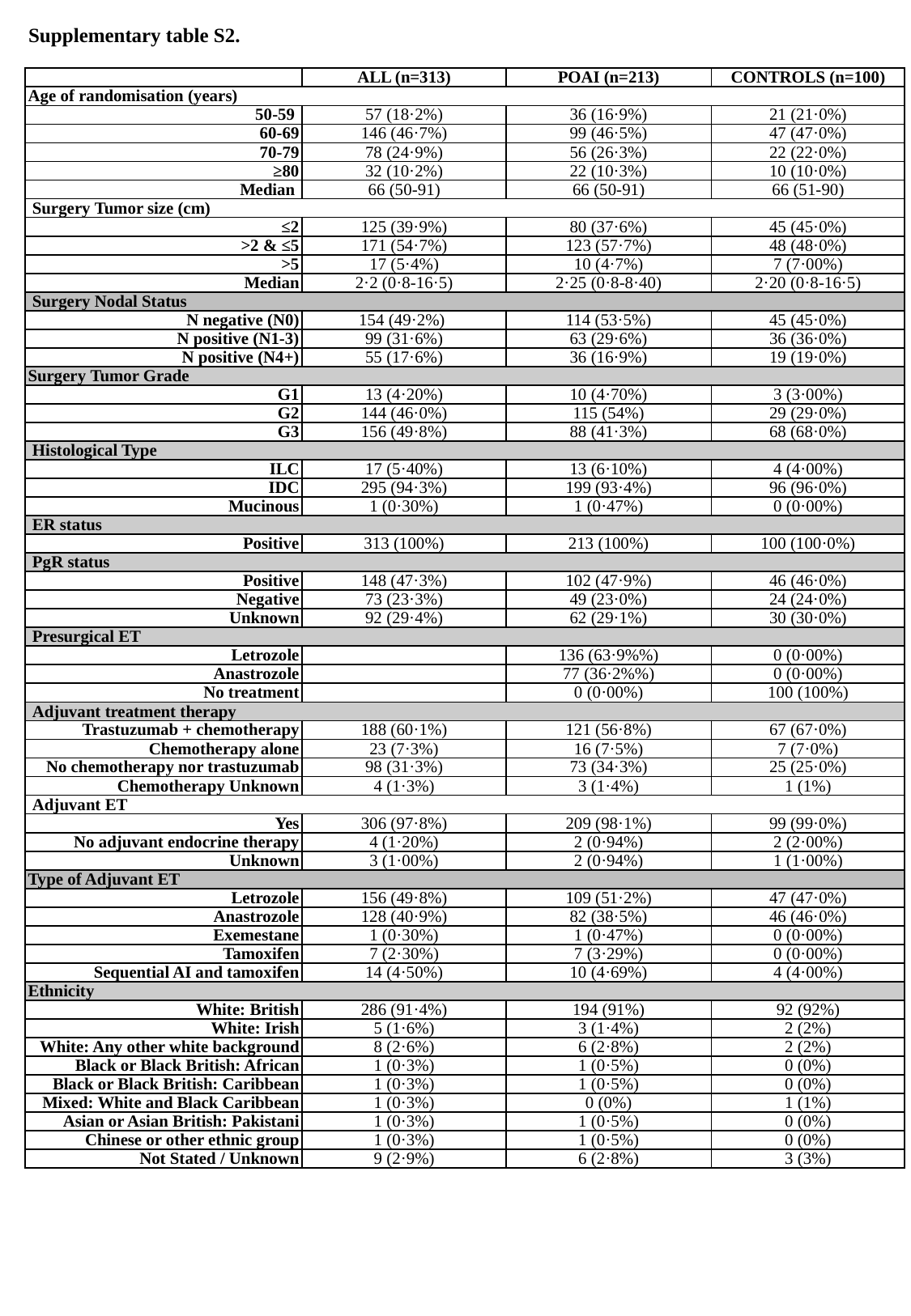

Supplementary table S2.
| | ALL (n=313) | POAI (n=213) | CONTROLS (n=100) |
| --- | --- | --- | --- |
| Age of randomisation (years) | | | |
| 50-59 | 57 (18·2%) | 36 (16·9%) | 21 (21·0%) |
| 60-69 | 146 (46·7%) | 99 (46·5%) | 47 (47·0%) |
| 70-79 | 78 (24·9%) | 56 (26·3%) | 22 (22·0%) |
| ≥80 | 32 (10·2%) | 22 (10·3%) | 10 (10·0%) |
| Median | 66 (50-91) | 66 (50-91) | 66 (51-90) |
| Surgery Tumor size (cm) | | | |
| ≤2 | 125 (39·9%) | 80 (37·6%) | 45 (45·0%) |
| >2 & ≤5 | 171 (54·7%) | 123 (57·7%) | 48 (48·0%) |
| >5 | 17 (5·4%) | 10 (4·7%) | 7 (7·00%) |
| Median | 2·2 (0·8-16·5) | 2·25 (0·8-8·40) | 2·20 (0·8-16·5) |
| Surgery Nodal Status | | | |
| N negative (N0) | 154 (49·2%) | 114 (53·5%) | 45 (45·0%) |
| N positive (N1-3) | 99 (31·6%) | 63 (29·6%) | 36 (36·0%) |
| N positive (N4+) | 55 (17·6%) | 36 (16·9%) | 19 (19·0%) |
| Surgery Tumor Grade | | | |
| G1 | 13 (4·20%) | 10 (4·70%) | 3 (3·00%) |
| G2 | 144 (46·0%) | 115 (54%) | 29 (29·0%) |
| G3 | 156 (49·8%) | 88 (41·3%) | 68 (68·0%) |
| Histological Type | | | |
| ILC | 17 (5·40%) | 13 (6·10%) | 4 (4·00%) |
| IDC | 295 (94·3%) | 199 (93·4%) | 96 (96·0%) |
| Mucinous | 1 (0·30%) | 1 (0·47%) | 0 (0·00%) |
| ER status | | | |
| Positive | 313 (100%) | 213 (100%) | 100 (100·0%) |
| PgR status | | | |
| Positive | 148 (47·3%) | 102 (47·9%) | 46 (46·0%) |
| Negative | 73 (23·3%) | 49 (23·0%) | 24 (24·0%) |
| Unknown | 92 (29·4%) | 62 (29·1%) | 30 (30·0%) |
| Presurgical ET | | | |
| Letrozole | | 136 (63·9%%) | 0 (0·00%) |
| Anastrozole | | 77 (36·2%%) | 0 (0·00%) |
| No treatment | | 0 (0·00%) | 100 (100%) |
| Adjuvant treatment therapy | | | |
| Trastuzumab + chemotherapy | 188 (60·1%) | 121 (56·8%) | 67 (67·0%) |
| Chemotherapy alone | 23 (7·3%) | 16 (7·5%) | 7 (7·0%) |
| No chemotherapy nor trastuzumab | 98 (31·3%) | 73 (34·3%) | 25 (25·0%) |
| Chemotherapy Unknown | 4 (1·3%) | 3 (1·4%) | 1 (1%) |
| Adjuvant ET | | | |
| Yes | 306 (97·8%) | 209 (98·1%) | 99 (99·0%) |
| No adjuvant endocrine therapy | 4 (1·20%) | 2 (0·94%) | 2 (2·00%) |
| Unknown | 3 (1·00%) | 2 (0·94%) | 1 (1·00%) |
| Type of Adjuvant ET | | | |
| Letrozole | 156 (49·8%) | 109 (51·2%) | 47 (47·0%) |
| Anastrozole | 128 (40·9%) | 82 (38·5%) | 46 (46·0%) |
| Exemestane | 1 (0·30%) | 1 (0·47%) | 0 (0·00%) |
| Tamoxifen | 7 (2·30%) | 7 (3·29%) | 0 (0·00%) |
| Sequential AI and tamoxifen | 14 (4·50%) | 10 (4·69%) | 4 (4·00%) |
| Ethnicity | | | |
| White: British | 286 (91·4%) | 194 (91%) | 92 (92%) |
| White: Irish | 5 (1·6%) | 3 (1·4%) | 2 (2%) |
| White: Any other white background | 8 (2·6%) | 6 (2·8%) | 2 (2%) |
| Black or Black British: African | 1 (0·3%) | 1 (0·5%) | 0 (0%) |
| Black or Black British: Caribbean | 1 (0·3%) | 1 (0·5%) | 0 (0%) |
| Mixed: White and Black Caribbean | 1 (0·3%) | 0 (0%) | 1 (1%) |
| Asian or Asian British: Pakistani | 1 (0·3%) | 1 (0·5%) | 0 (0%) |
| Chinese or other ethnic group | 1 (0·3%) | 1 (0·5%) | 0 (0%) |
| Not Stated / Unknown | 9 (2·9%) | 6 (2·8%) | 3 (3%) |

## Slide 5
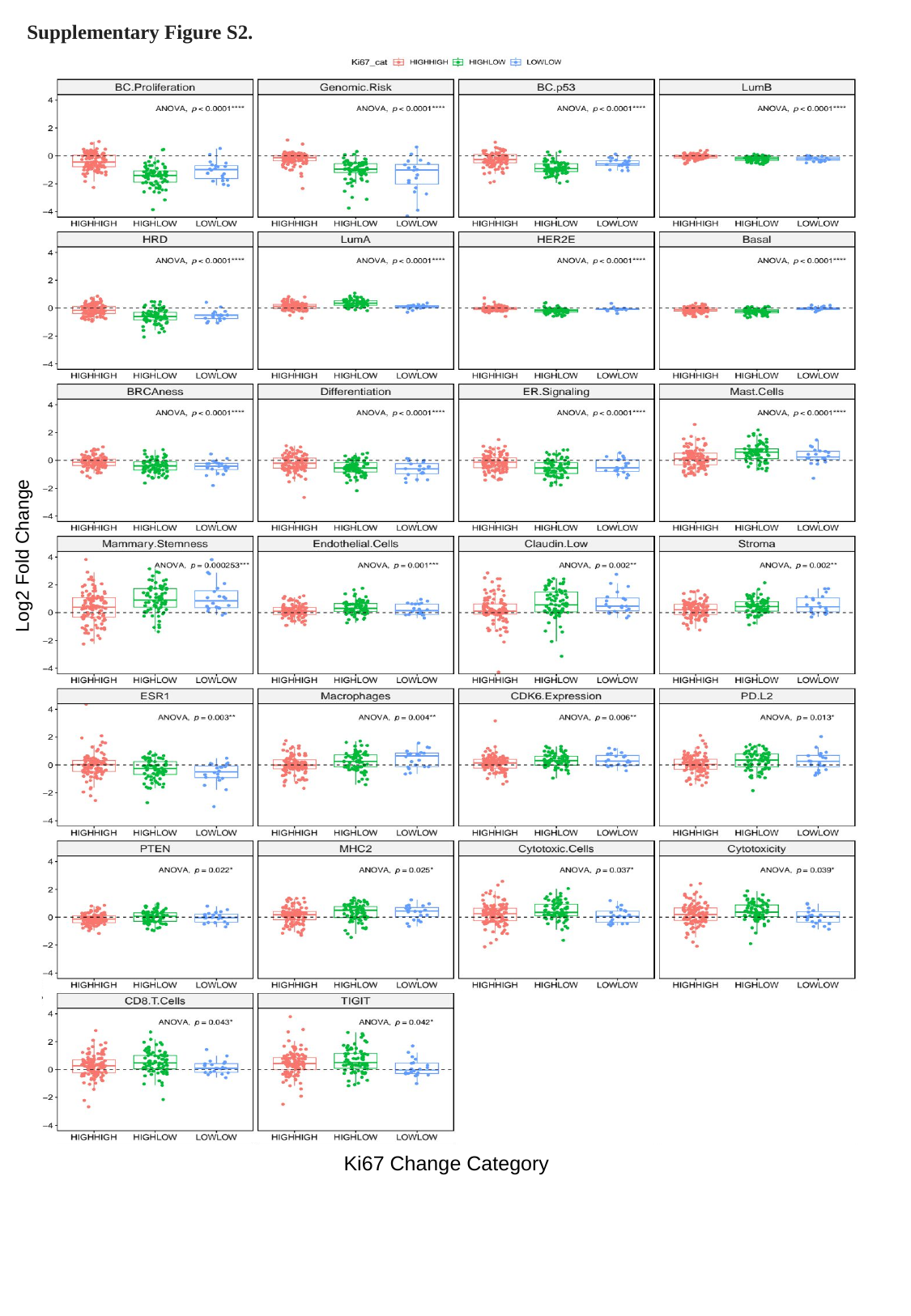

Supplementary Figure S2.
Log2 Fold Change
Ki67 Change Category

## Slide 6
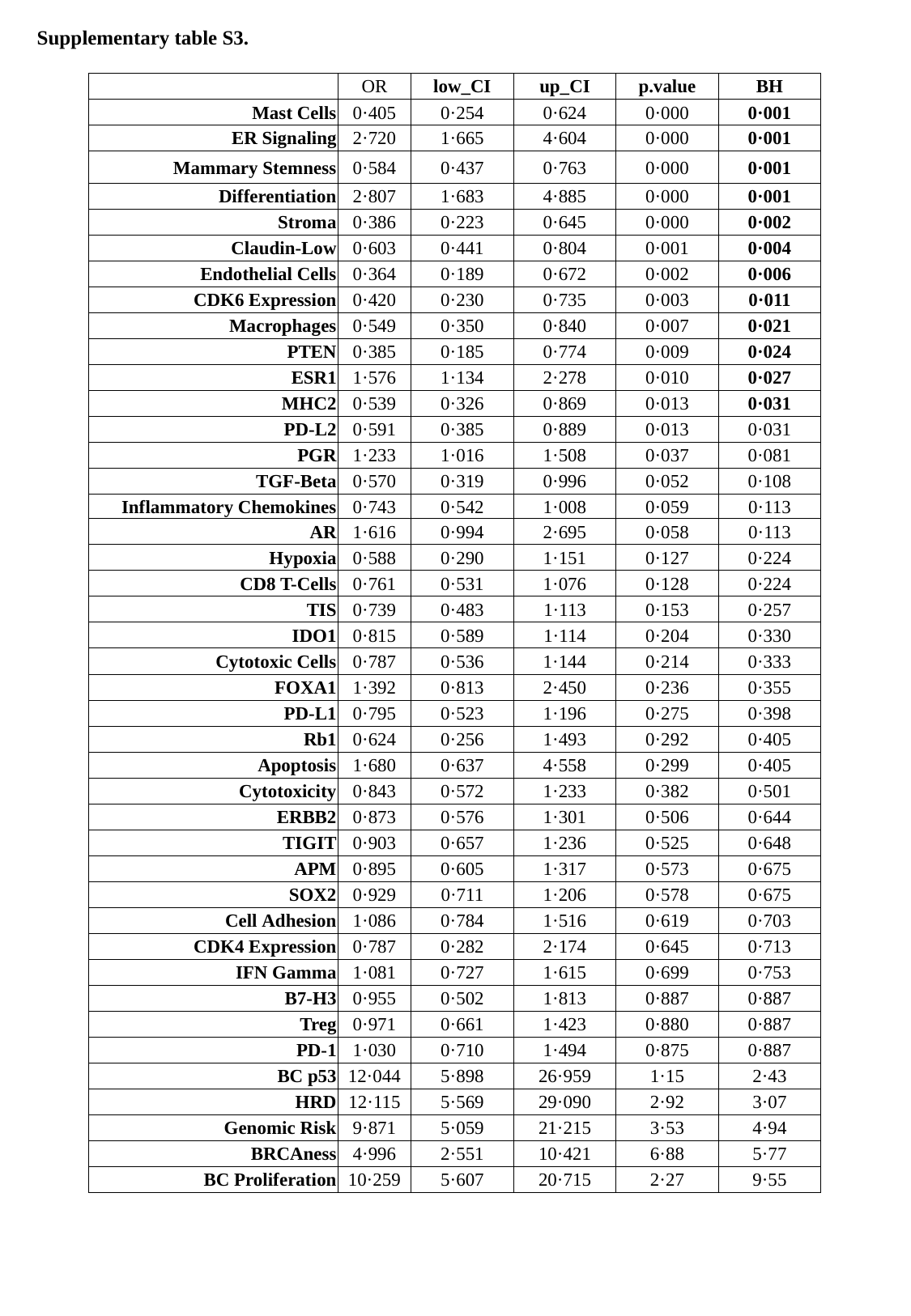

Supplementary table S3.
| | OR | low\_CI | up\_CI | p.value | BH |
| --- | --- | --- | --- | --- | --- |
| Mast Cells | 0·405 | 0·254 | 0·624 | 0·000 | 0·001 |
| ER Signaling | 2·720 | 1·665 | 4·604 | 0·000 | 0·001 |
| Mammary Stemness | 0·584 | 0·437 | 0·763 | 0·000 | 0·001 |
| Differentiation | 2·807 | 1·683 | 4·885 | 0·000 | 0·001 |
| Stroma | 0·386 | 0·223 | 0·645 | 0·000 | 0·002 |
| Claudin-Low | 0·603 | 0·441 | 0·804 | 0·001 | 0·004 |
| Endothelial Cells | 0·364 | 0·189 | 0·672 | 0·002 | 0·006 |
| CDK6 Expression | 0·420 | 0·230 | 0·735 | 0·003 | 0·011 |
| Macrophages | 0·549 | 0·350 | 0·840 | 0·007 | 0·021 |
| PTEN | 0·385 | 0·185 | 0·774 | 0·009 | 0·024 |
| ESR1 | 1·576 | 1·134 | 2·278 | 0·010 | 0·027 |
| MHC2 | 0·539 | 0·326 | 0·869 | 0·013 | 0·031 |
| PD-L2 | 0·591 | 0·385 | 0·889 | 0·013 | 0·031 |
| PGR | 1·233 | 1·016 | 1·508 | 0·037 | 0·081 |
| TGF-Beta | 0·570 | 0·319 | 0·996 | 0·052 | 0·108 |
| Inflammatory Chemokines | 0·743 | 0·542 | 1·008 | 0·059 | 0·113 |
| AR | 1·616 | 0·994 | 2·695 | 0·058 | 0·113 |
| Hypoxia | 0·588 | 0·290 | 1·151 | 0·127 | 0·224 |
| CD8 T-Cells | 0·761 | 0·531 | 1·076 | 0·128 | 0·224 |
| TIS | 0·739 | 0·483 | 1·113 | 0·153 | 0·257 |
| IDO1 | 0·815 | 0·589 | 1·114 | 0·204 | 0·330 |
| Cytotoxic Cells | 0·787 | 0·536 | 1·144 | 0·214 | 0·333 |
| FOXA1 | 1·392 | 0·813 | 2·450 | 0·236 | 0·355 |
| PD-L1 | 0·795 | 0·523 | 1·196 | 0·275 | 0·398 |
| Rb1 | 0·624 | 0·256 | 1·493 | 0·292 | 0·405 |
| Apoptosis | 1·680 | 0·637 | 4·558 | 0·299 | 0·405 |
| Cytotoxicity | 0·843 | 0·572 | 1·233 | 0·382 | 0·501 |
| ERBB2 | 0·873 | 0·576 | 1·301 | 0·506 | 0·644 |
| TIGIT | 0·903 | 0·657 | 1·236 | 0·525 | 0·648 |
| APM | 0·895 | 0·605 | 1·317 | 0·573 | 0·675 |
| SOX2 | 0·929 | 0·711 | 1·206 | 0·578 | 0·675 |
| Cell Adhesion | 1·086 | 0·784 | 1·516 | 0·619 | 0·703 |
| CDK4 Expression | 0·787 | 0·282 | 2·174 | 0·645 | 0·713 |
| IFN Gamma | 1·081 | 0·727 | 1·615 | 0·699 | 0·753 |
| B7-H3 | 0·955 | 0·502 | 1·813 | 0·887 | 0·887 |
| Treg | 0·971 | 0·661 | 1·423 | 0·880 | 0·887 |
| PD-1 | 1·030 | 0·710 | 1·494 | 0·875 | 0·887 |
| BC p53 | 12·044 | 5·898 | 26·959 | 1·15 | 2·43 |
| HRD | 12·115 | 5·569 | 29·090 | 2·92 | 3·07 |
| Genomic Risk | 9·871 | 5·059 | 21·215 | 3·53 | 4·94 |
| BRCAness | 4·996 | 2·551 | 10·421 | 6·88 | 5·77 |
| BC Proliferation | 10·259 | 5·607 | 20·715 | 2·27 | 9·55 |

## Slide 7
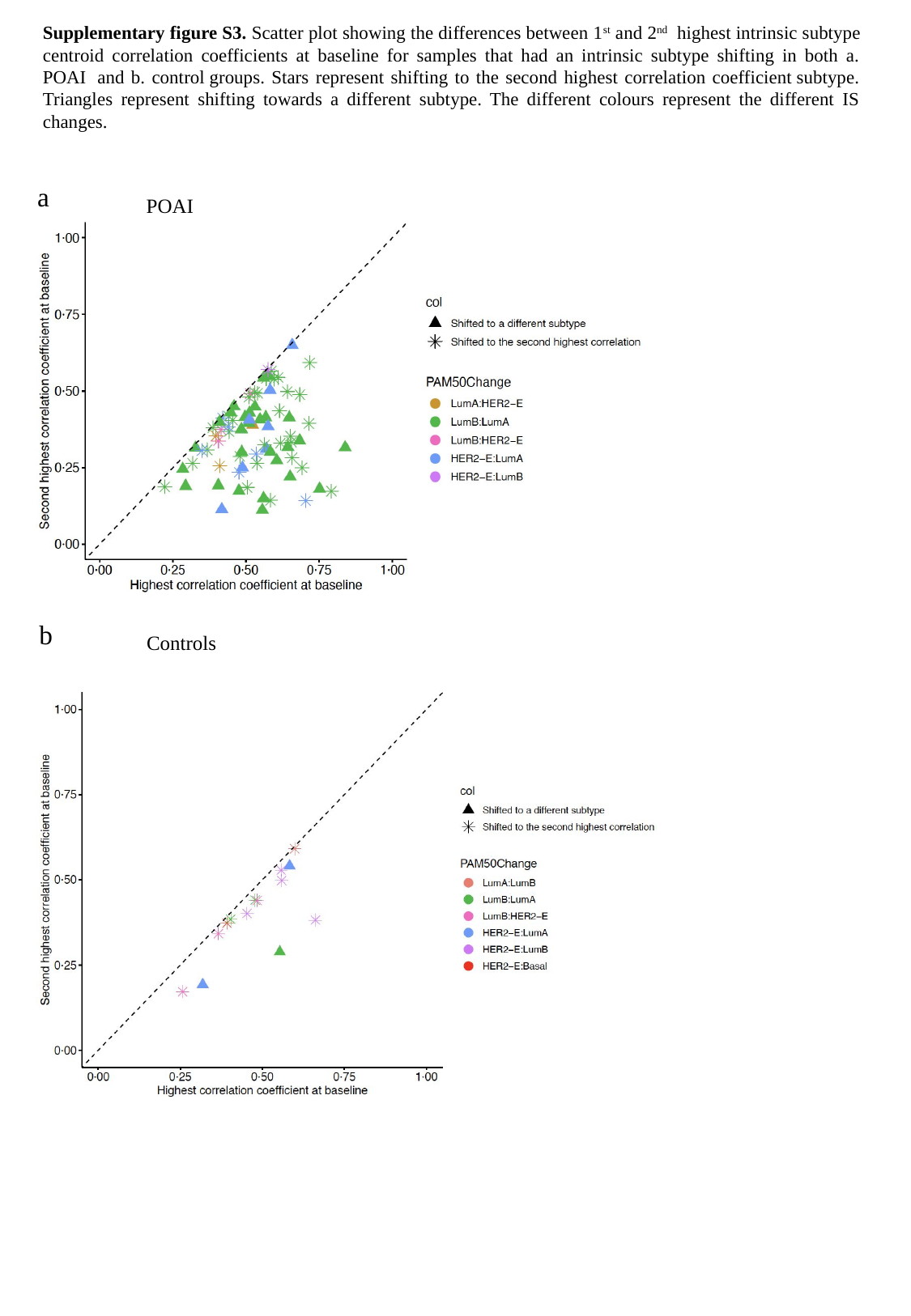

Supplementary figure S3. Scatter plot showing the differences between 1st and 2nd  highest intrinsic subtype centroid correlation coefficients at baseline for samples that had an intrinsic subtype shifting in both a. POAI  and b. control groups. Stars represent shifting to the second highest correlation coefficient subtype. Triangles represent shifting towards a different subtype. The different colours represent the different IS changes.
a
POAI
b
Controls

## Slide 8
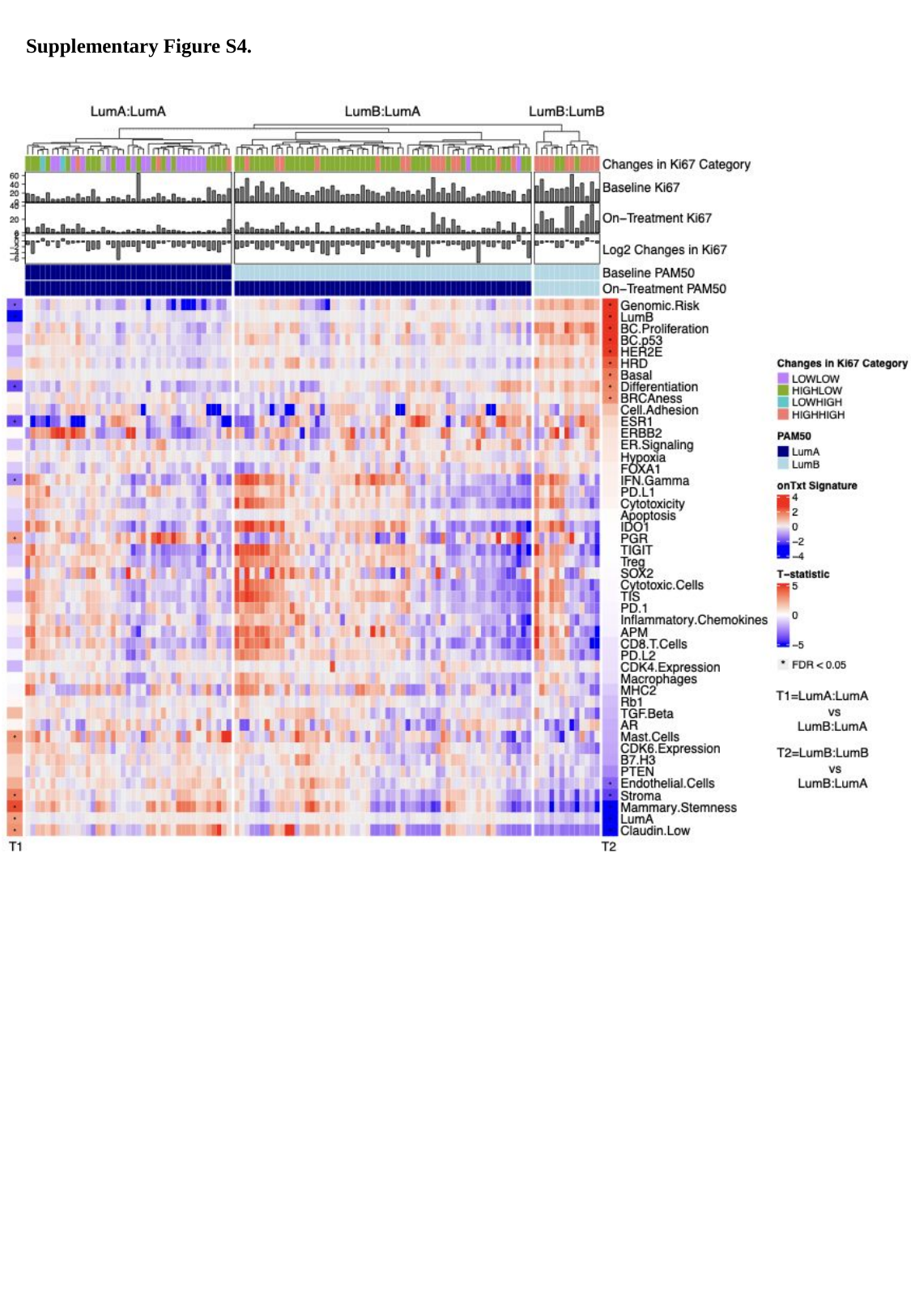

Supplementary Figure S4.

## Slide 9
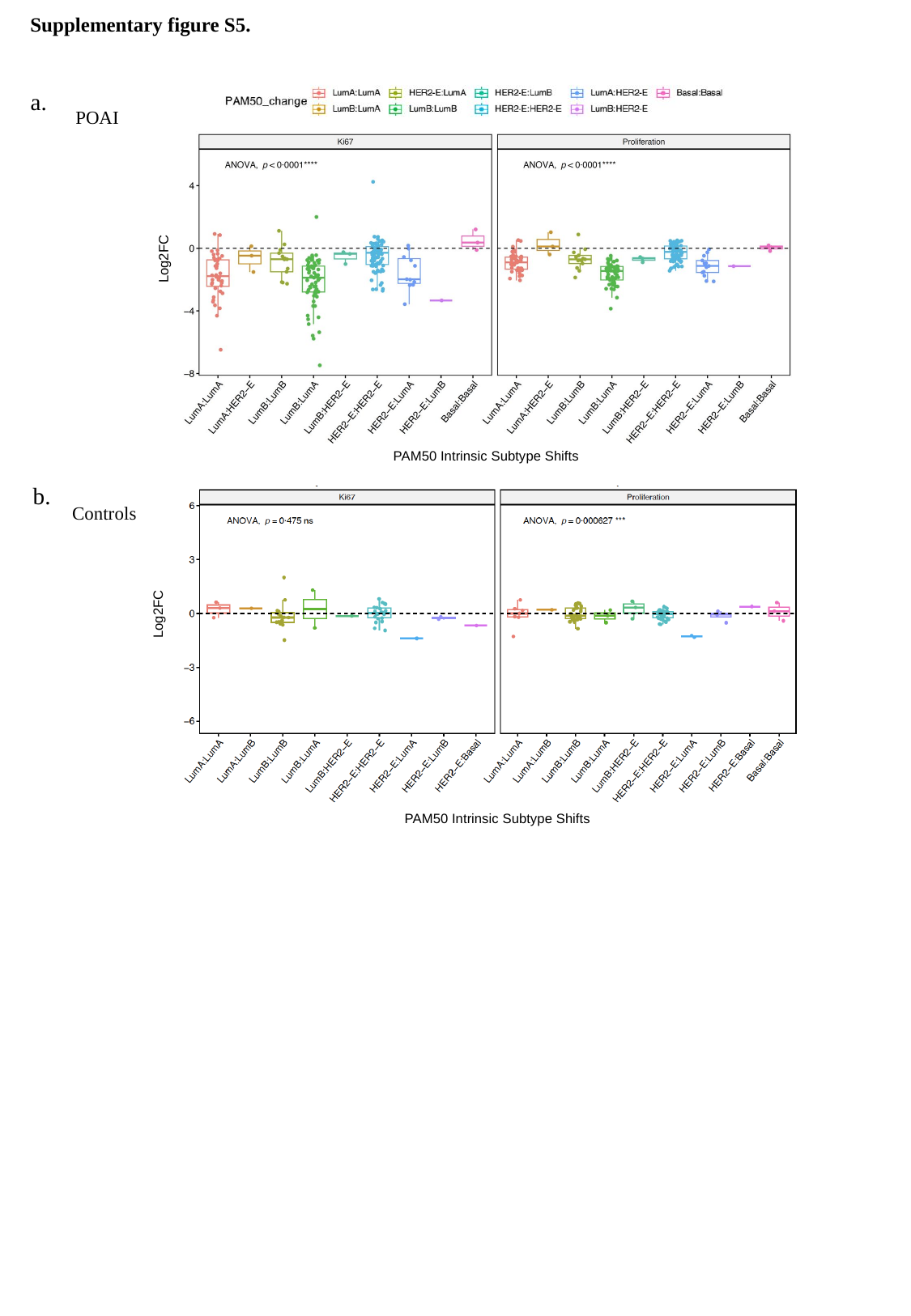

Supplementary figure S5.
a.
POAI
Log2FC
Log2FC
PAM50 Intrinsic Subtype Shifts
b.
Controls
Log2FC
PAM50 Intrinsic Subtype Shifts

## Slide 10
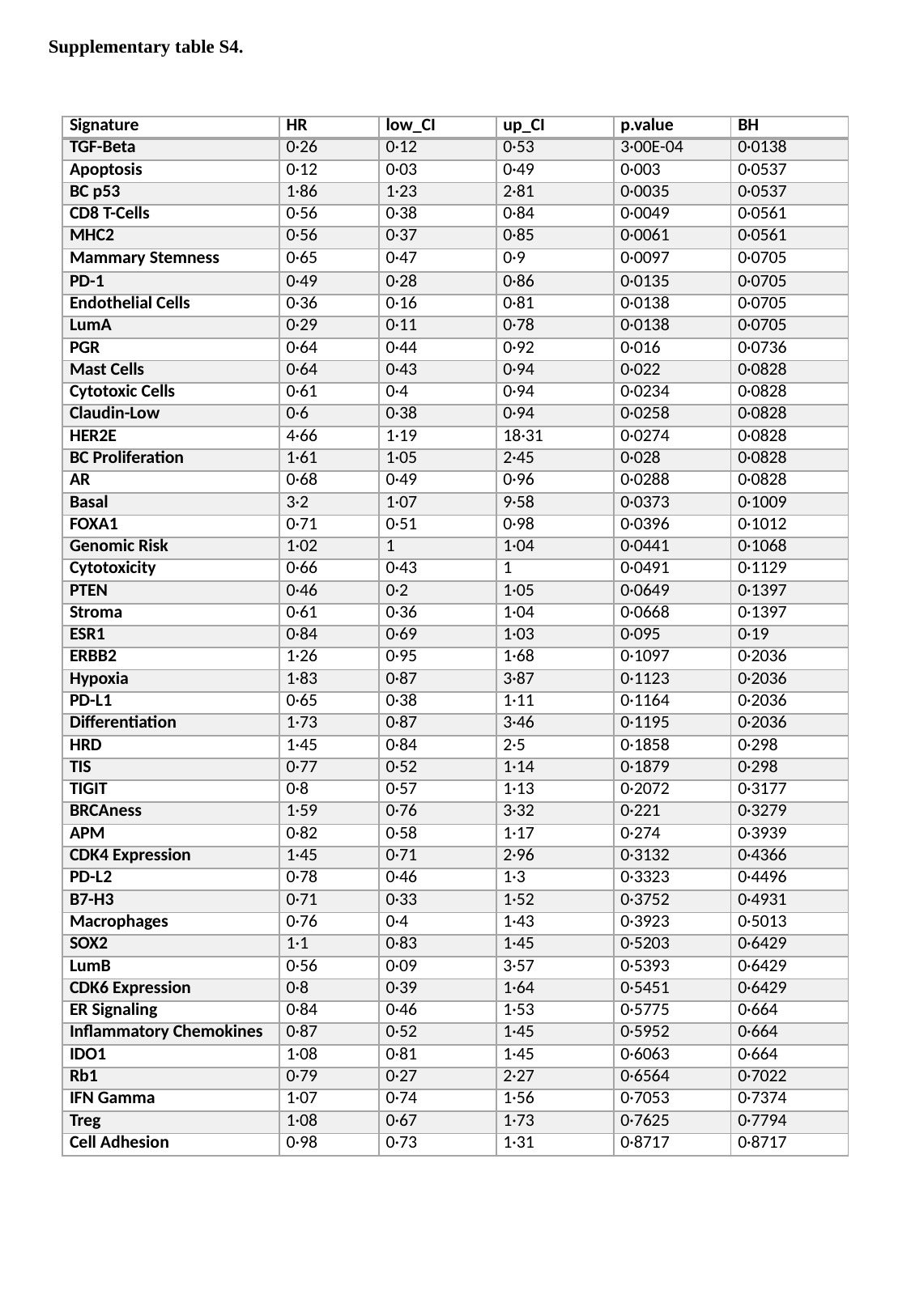

Supplementary table S4.
| Signature | HR | low\_CI | up\_CI | p.value | BH |
| --- | --- | --- | --- | --- | --- |
| TGF-Beta | 0·26 | 0·12 | 0·53 | 3·00E-04 | 0·0138 |
| Apoptosis | 0·12 | 0·03 | 0·49 | 0·003 | 0·0537 |
| BC p53 | 1·86 | 1·23 | 2·81 | 0·0035 | 0·0537 |
| CD8 T-Cells | 0·56 | 0·38 | 0·84 | 0·0049 | 0·0561 |
| MHC2 | 0·56 | 0·37 | 0·85 | 0·0061 | 0·0561 |
| Mammary Stemness | 0·65 | 0·47 | 0·9 | 0·0097 | 0·0705 |
| PD-1 | 0·49 | 0·28 | 0·86 | 0·0135 | 0·0705 |
| Endothelial Cells | 0·36 | 0·16 | 0·81 | 0·0138 | 0·0705 |
| LumA | 0·29 | 0·11 | 0·78 | 0·0138 | 0·0705 |
| PGR | 0·64 | 0·44 | 0·92 | 0·016 | 0·0736 |
| Mast Cells | 0·64 | 0·43 | 0·94 | 0·022 | 0·0828 |
| Cytotoxic Cells | 0·61 | 0·4 | 0·94 | 0·0234 | 0·0828 |
| Claudin-Low | 0·6 | 0·38 | 0·94 | 0·0258 | 0·0828 |
| HER2E | 4·66 | 1·19 | 18·31 | 0·0274 | 0·0828 |
| BC Proliferation | 1·61 | 1·05 | 2·45 | 0·028 | 0·0828 |
| AR | 0·68 | 0·49 | 0·96 | 0·0288 | 0·0828 |
| Basal | 3·2 | 1·07 | 9·58 | 0·0373 | 0·1009 |
| FOXA1 | 0·71 | 0·51 | 0·98 | 0·0396 | 0·1012 |
| Genomic Risk | 1·02 | 1 | 1·04 | 0·0441 | 0·1068 |
| Cytotoxicity | 0·66 | 0·43 | 1 | 0·0491 | 0·1129 |
| PTEN | 0·46 | 0·2 | 1·05 | 0·0649 | 0·1397 |
| Stroma | 0·61 | 0·36 | 1·04 | 0·0668 | 0·1397 |
| ESR1 | 0·84 | 0·69 | 1·03 | 0·095 | 0·19 |
| ERBB2 | 1·26 | 0·95 | 1·68 | 0·1097 | 0·2036 |
| Hypoxia | 1·83 | 0·87 | 3·87 | 0·1123 | 0·2036 |
| PD-L1 | 0·65 | 0·38 | 1·11 | 0·1164 | 0·2036 |
| Differentiation | 1·73 | 0·87 | 3·46 | 0·1195 | 0·2036 |
| HRD | 1·45 | 0·84 | 2·5 | 0·1858 | 0·298 |
| TIS | 0·77 | 0·52 | 1·14 | 0·1879 | 0·298 |
| TIGIT | 0·8 | 0·57 | 1·13 | 0·2072 | 0·3177 |
| BRCAness | 1·59 | 0·76 | 3·32 | 0·221 | 0·3279 |
| APM | 0·82 | 0·58 | 1·17 | 0·274 | 0·3939 |
| CDK4 Expression | 1·45 | 0·71 | 2·96 | 0·3132 | 0·4366 |
| PD-L2 | 0·78 | 0·46 | 1·3 | 0·3323 | 0·4496 |
| B7-H3 | 0·71 | 0·33 | 1·52 | 0·3752 | 0·4931 |
| Macrophages | 0·76 | 0·4 | 1·43 | 0·3923 | 0·5013 |
| SOX2 | 1·1 | 0·83 | 1·45 | 0·5203 | 0·6429 |
| LumB | 0·56 | 0·09 | 3·57 | 0·5393 | 0·6429 |
| CDK6 Expression | 0·8 | 0·39 | 1·64 | 0·5451 | 0·6429 |
| ER Signaling | 0·84 | 0·46 | 1·53 | 0·5775 | 0·664 |
| Inflammatory Chemokines | 0·87 | 0·52 | 1·45 | 0·5952 | 0·664 |
| IDO1 | 1·08 | 0·81 | 1·45 | 0·6063 | 0·664 |
| Rb1 | 0·79 | 0·27 | 2·27 | 0·6564 | 0·7022 |
| IFN Gamma | 1·07 | 0·74 | 1·56 | 0·7053 | 0·7374 |
| Treg | 1·08 | 0·67 | 1·73 | 0·7625 | 0·7794 |
| Cell Adhesion | 0·98 | 0·73 | 1·31 | 0·8717 | 0·8717 |

## Slide 11
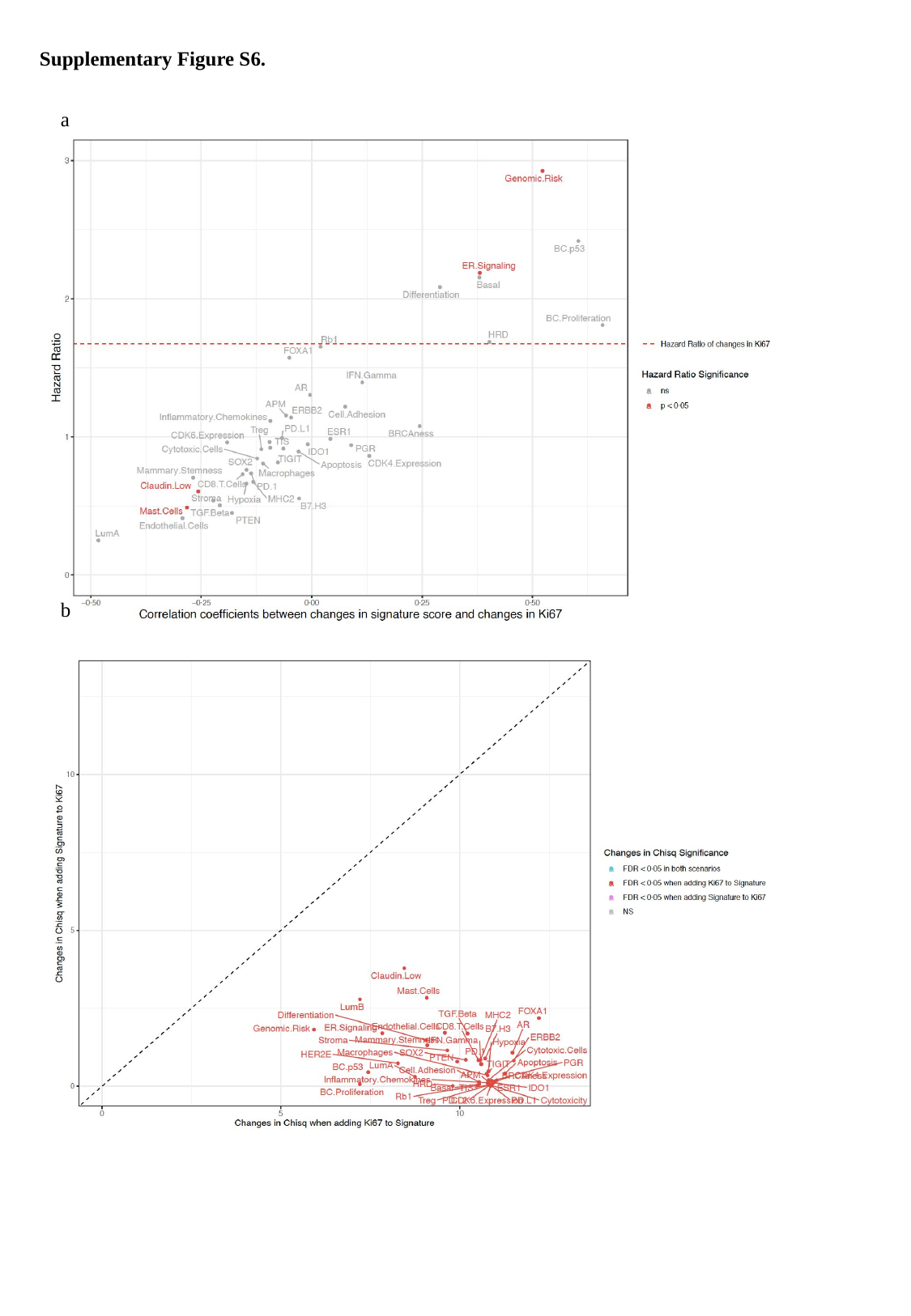

Supplementary Figure S6.
a
b
