# Supplementary material for: Short-duration preoperative endocrine therapy alters molecular profiles to predict favourable outcome in ER+/HER2+ early breast cancer: a POETIC translational study
Source: eBioMedicine. 2025 Jul 18;118:105823. doi: 10.1016/j.ebiom.2025.105823 (PMC12368290; doi:10.1016/j.ebiom.2025.105823)
Supplement: POETIC Protocol Version6 [file mmc2.pdf]

Poetic

Trial of Perioperative Endocrine Therapy - Individualising Care

Chief Investigator: Professor Ian Smith

Co-Sponsors: The Royal Marsden NHS Foundation Trust
The Institute of Cancer Research

Approval: Clinical Trials Advisory & Awards Committee (CTAAC)
Translational Research in Clinical Trials (TRICC)

Funders: Cancer Research UK

Coordinating Clinical Trials Unit: The Institute of Cancer Research Clinical Trials and Statistics Unit (ICR-CTSU)

Endorsements: Association of Breast Surgery at BASO

Version 6 – 8 July 2015

This trial is part of the National Institute for Health Research (NIHR) portfolio as a high priority trial.

Main REC Reference Number: 08/H1102/37
ISRCTN: 63882543
EudraCT Number: 2007-003877-21
CRUK Number: CRUK/07/015
CTA Number: 22138/0005/001-0001
ICR/RMH CCR Number: 2973
ICR-CTSU Protocol Number: 2007/10015

TRIAL ADMINISTRATION

CLINICAL COORDINATION

Professor Ian Smith (Chief Investigator)
Royal Marsden NHS Foundation Trust
Fulham Road
London SW3 6JJ
Tel: 020 7808 2751
Fax: 020 7352 5441
Email: Ian.Smith@rmh.nhs.uk

John Robertson (Surgical Lead)
School of Graduate Entry Medicine & Health (GEM), Royal Derby Hospital, Uttoxeter Road
Derby DE22 3DT
Tel: 01332 724881
Fax: 0115 8231877
Email: John.Robertson@nottingham.ac.uk

Mitch Dowsett (Biological Studies Lead)
Department of Academic Biochemistry
Royal Marsden NHS Foundation Trust
Fulham Road
London SW3 6JJ
Tel: 020 7808 2887
Email: Mitch.Dowsett@icr.ac.uk

ICR-CTSU (an NCRI accredited clinical trials unit)
has overall responsibility for the conduct of the trial.

ICR-CTSU
Division of Clinical Studies
The Institute of Cancer Research
Sir Richard Doll Building
Cotswold Road
Sutton, Surrey SM2 5NG

POETIC Trial Manager: Jane Banerji
Tel: 020 8722 4349
Email: poetic-icrctsu@icr.ac.uk

Senior Trials Manager: Monique Tomiczek
Tel: 020 8722 4000 ext. 4808
Email: Monique.Tomiczek@icr.ac.uk

Translational Scientist: Maggie Cheang
Tel: 020 8722 4552
Email: Maggie.Cheang@icr.ac.uk

Statistician: James Morden
Tel: 020 8722 4187
Email: James.Morden@icr.ac.uk

ICR-CTSU Scientific Lead: Judith Bliss
Tel: 020 8722 4297 / 4013
Email: Judith.Bliss@icr.ac.uk

Any questions relating to this protocol should be addressed in the first instance to the POETIC Trial Manager within ICR-CTSU:

Email: poetic-icrctsu@icr.ac.uk

General enquiries: 0208 722 4185/4157
Fax: 0208 770 7876

Randomisation line: +44 (0)20 8643 7150
09:00 – 17:00 Monday-Friday

Protocol Development Group: *Roger A'Hern (Sutton), Hugh Bishop (Bolton), Judith Bliss (Sutton), Nigel J Bundred (Manchester), Robert Carpenter (London), Robert Coleman (Sheffield), Erika Denton (Norwich), John Dewar (Dundee), Mike Dixon (Edinburgh), Julie Doughty (Glasgow), Mitch Dowsett (London), Lindsay Johnson (Sutton), Elizabeth Mallon (Glasgow), Rob Nicholson (Cardiff), Robin Prescott (Edinburgh), Arnie Purusotham (London), John Robertson (Nottingham), Ian Smith (London), Rosemary Walker (Leicester), Robin Wilson (London)*

The Trial Management Group (TMG) is constituted from members of the Protocol Development Group and selected principal investigators from participating centres. A copy of the current membership of the TMG can be obtained from the POETIC Trial manager within ICR-CTSU.

Protocol Authorised by:

Name & Role:	Signature:	Date:
Professor Ian Smith	[redacted]	08 July 2015

This protocol describes the POETIC trial and provides information about procedures for entering patients. The protocol should not be used as a guide for the treatment of other patients; every care was taken in its preparation, but corrections or amendments may be necessary. These will be circulated to investigators in the trial, but centres entering patients for the first time are advised to contact The Institute of Cancer Research - Clinical Trials & Statistics Unit (ICR-CTSU) to confirm they have the most recent version. Protocol amendments will be circulated to participating centres as they occur.

This protocol is a controlled document and should not be copied, distributed or reproduced without the written permission of the ICR-CTSU.

Queries relating to this trial should be referred, in the first instance, to the POETIC Trial Manager within ICR-CTSU.

This trial will adhere to the principles outlined in the Medicines for Human Use (Clinical Trials) Regulations 2004 (SI 2004/1031). It will be conducted in compliance with the protocol, the Data Protection Act (Z6364106) and other regulatory requirements as appropriate.

TABLE OF CONTENTS

1. Trial Summary 1

2. Introduction 2

3. Background and Rationale 2

3.1 Consultation on trial procedures and conduct 3

4. Trial Objectives 4

4.1 Exploratory Objectives: 5

5. Study Design 6

6. Endpoints 8

6.1 Primary endpoints: 8

6.2 Secondary endpoints: 8

6.3 Exploratory Endpoints 8

7. Patient Selection and Eligibility 8

7.1 Source of patients 8

7.2 Number of patients 8

7.3 Inclusion criteria: 8

7.4 Exclusion criteria: 9

8. Procedure for Obtaining Consent for Trial Entry & Tissue Donation 9

9. Biological Specimen Collection 12

9.1 Specimen Collection 12

9.2 Use of tissue sample collection beyond POETIC 13

10. Randomisation Procedure 13

11. Trial Treatment 14

12. Drug supplies & labelling 15

13. Non Trial Treatment 15

13.1 Surgical Treatment 15

13.2 Radiotherapy 15

13.3 Adjuvant Endocrine therapy 15

13.4 Adjuvant Chemotherapy 16

13.5 Adjuvant Herceptin and Bisphosphonates 16

14. Trial Evaluations 16

14.1 Staging investigations 16

14.2 Follow–up Investigations 16

15. Pharmacovigilance 16

15.1 Definitions 16

15.1.1 Serious Adverse Events (SAEs) 16

15.1.2 Serious Adverse Reactions (SAR) 17

15.1.3 Suspected Unexpected Serious Adverse Reactions (SUSARs) 17

15.2 Causality (relatedness) 17

15.3 Reporting of Serious Adverse Events/Reactions to ICR-CTSU 18

15.4 Recording and Reporting of Serious Adverse Events 18

15.5 Review of Serious Adverse Event 18

15.6 Expedited Reporting of SUSARs 18

15.7 Follow-up of Serious Adverse Events 19

15.8 Annual Reporting of Serious Adverse Reactions 19

16. Statistical Considerations 21

16.1 Trial Hypothesis 21

16.2 Stratification 21

16.3 Randomisation 21

16.4 Sample Size 21

16.5 Analysis Plan 22

16.6 Interim Analyses and role of Independent Data Monitoring Committee (IDMC) 23

17. Study organisation 24

17.1 Data monitoring plan 24

18. Follow-up management and completion of Case Report Forms (CRFs) 24

18.1 Follow-up 24

18.2 Relapse 24

19. Patient Protections and Ethical considerations 25

19.1 Liability/Indemnity/Insurance 25

19.2 Patient Confidentiality 25

20. Withdrawal of patients from the trial treatment 26

20.1 Withdrawal of patients from trial treatment 26

20.2 Withdrawal of patients from trial follow-up 26

21. Completion of the study and definition of study end date 26

22. Research Governance 26

22.1 Trial Administration 26

22.1.1 Trial Administration - Royal Marsden Hospital Responsibilities 26

22.1.2 Trial Administration - The Institute of Cancer Research Responsibilities 27

22.2 Protocol Compliance & Initiation 28

22.3 Data Acquisition & On-Site Monitoring 28

22.4 Archiving 29

22.5 Data Protection Act (DPA) 29

22.6 Financial Matters 29

22.7 Clinical risk assessment 29

23. Publication policy 30

24. References 30

Appendix 1 Glossary of Terms 32

Appendix 2 - Patient Pathways 33

Appendix 3 – Tissue sample collection and analysis 35

A. Sample collection: 35

Appendix 4 – WHO Performance Status 37

Appendix 5 - Patient information sheet and consent forms 37

1. Trial Summary

**TITLE:**	Trial of Perioperative Endocrine Therapy – Individualising Care (POETIC)
**OBJECTIVES:**	To determine whether perioperative endocrine therapy with an aromatase inhibitor (AI) followed by standard adjuvant therapy improves outcome compared with standard adjuvant therapy alone in postmenopausal women with hormone receptor positive breast cancer.<br><br>To determine whether the proliferation marker Ki67 as measured by immunohistochemistry (IHC) in the excised cancer around 2 weeks after starting AI therapy will predict for time to recurrence (TTR) in the individual patient more effectively than the pre-treatment Ki67 value.<br><br>To determine whether molecular profiling 2 weeks after starting endocrine therapy predicts for long-term outcome in postmenopausal women with hormone receptor positive breast cancer better than at diagnosis.
**Exploratory Objectives:**	To deconstruct the underlying mechanisms that might explain the development of metastatic disease by correlating the genomic data of primary and metastatic tissue a) with clinical data of patients and b) against Ki67 and other molecular features measured at baseline and 2-week post-treatment.<br><br>To determine whether the amount of ctDNA in the plasma collected could act as a measure of residual disease after surgery and predictor of relapse.
**TRIAL DESIGN:**	Phase III, multi-centre, randomised trial.<br><br>Patients will be allocated in a 2:1 ratio to PERIOPERATIVE THERAPY with an AI for 4 weeks (two weeks before and two weeks after surgery) or NO PERIOPERATIVE THERAPY.
**PATIENT TYPE/NUMBER:**	Postmenopausal women with ER/PgR positive primary breast cancer.<br><br>The trial aims to recruit approximately 4350 patients plus the number required to complete POETIC sub-studies.
**TRIAL TREATMENT:**	Group I: PERIOPERATIVE THERAPY with an AI<br><br>Choice of AI is according to centre policy and may be either anastrozole (1mg/day) or letrozole (2.5mg/day)<br><br>Group II: NO PERIOPERATIVE THERAPY
**TREATMENT DURATION:**	4 weeks (two weeks before and two weeks after surgery)
**ENDPOINTS:**	**Primary endpoints:**<br>• Time to recurrence (clinical endpoint)<br><br>**Secondary endpoints:**<br>• Relapse free survival

- Time to local recurrence
- Time to distant recurrence
- Overall survival
- Breast cancer free survival
- Proliferation rate (Ki67) at baseline core biopsy and at surgical excision (biological endpoint)Gene expression profile at core biopsy, and at surgical excision

Exploratory endpoints

- Gene expression profile at metastatic excision;
- Mutation profiles at core biopsy, surgical and metastatic excision and
- Somatic DNA alterations of ctDNA that might be defined as coming from the primary tumour

2. Introduction

Experimental evidence suggests that endocrine therapy shortly before and immediately after breast cancer surgery might improve outcome (1). This important hypothesis has never been tested clinically. Adjuvant endocrine therapy and adjuvant chemotherapy each result in significant survival benefits in women with early breast cancer (2), but techniques to predict benefit for the individual patient are very poorly developed, and treatment is planned on the basis of probability of the potential to achieve a worthwhile benefit, using standard prognostic and predictive parameters (size, grade, nodal involvement, hormone receptor status, HER2 status and age). A means of predicting outcome of endocrine therapy with molecular techniques in an individual patient would allow much more rational decisions on the need for additional therapy than is currently possible. This would minimise unnecessary toxicity, save on resources, and hopefully improve outcome.

The POETIC trial aims to test these hypotheses in a randomised clinical trial in postmenopausal patients with hormone receptor-positive breast cancer.

3. Background and Rationale

The rationale for systemic perioperative therapy is supported by the fact that access of tumour cells to the circulation is an essential step towards metastasis. A study of 18 patients detected circulating tumour cells in one patient before, in six during, and none after primary breast cancer surgery (3). The clinical significance of this is uncertain, but experimental systems indicate that non-curative reduction of tumour cell burden results in a more rapid doubling time of residual tumour (4). This increase in proliferation rate can be blocked by preoperative tamoxifen (1). More recent clinical work has indicated that two weeks preoperative therapy with an aromatase inhibitor (AI) or tamoxifen markedly reduces proliferation as measured by Ki67 in human breast cancer (5). These observations suggest that short duration perioperative endocrine therapy might improve long term outcome with no additional toxicity or resource implications. No major clinical trial has so far tested this hypothesis.

The relationship between baseline and two week Ki67 levels and long term outcome was recently analysed in the IMPACT trial comparing neoadjuvant anastrozole with tamoxifen with the combination, and using Ki67 as a primary endpoint (6). It was found that in 158 patients with a median follow-up of 37 months (maximum 88 months) both baseline and 2 week Ki67 levels were significantly related to relapse free survival but the relationship was stronger for 2 week Ki67 (p=0.008). Multivariate analysis showed a highly significant relationship between 2 week but not baseline Ki67 and relapse free survival (7). If these results can be confirmed, then it provides the opportunity for Ki67 measurement in the excised breast cancer in an individual patient treated with 2 weeks preoperative endocrine therapy to contribute to the assessment of the likely outcome in that patient. This would aid greatly in deciding whether additional adjuvant chemotherapy was required for that patient.

Advances in molecular techniques applied to human tissues have allowed the creation of numerous molecular signatures which appear to predict clinical outcome better than standard clinical-pathological markers (8). All of these profiles for breast cancer have been developed on untreated tumours yet they generally attempt to predict the outcome of patients who are then treated with a medical intervention, most frequently endocrine therapy. Two large trials to test the application of different molecular profiles are about to be initiated: MINDACT (9) and TAILORx (10). We have recently demonstrated however that the molecular profile of tumours treated with AIs is radically altered with the expression of over 2,000 (median) genes being significantly changed with 2 weeks treatment (11). These changes vary markedly between patients and it is rational that this may reflect the benefit derived from therapy. Thus it is possible that the recent data indicating that Ki67 measured after 2 weeks preoperative AI treatment may aid in predicting outcome on adjuvant endocrine therapy more accurately than Ki67 measured prior to AI treatment may be extendable to such molecular profiling. At the Royal Marsden Hospital, we have available c.100 pairs of tumour biopsies from ER+ patients taken from patients receiving anastrozole in our recently reported anastrozole±gefitinib neoadjuvant trial to extend our molecular findings to assist in the creation of an on-treatment molecular predictive profile (12). The molecular profile will be developed whilst recruitment to POETIC is ongoing. Samples will be taken from patients in POETIC to allow subsequent extension and validation of the profile.

In the absence of diagnostic tools that characterise whether the disease has been completely eradicated by surgery, the promising approach of circulating tumour DNA measurement as a personalised marker of minimal residual disease will be assessed

Acknowledging the importance of unravelling the mechanisms underlying breast cancer metastasis, gene molecular profile at metastasis against pre-treatment and post-treatment data will be studied.

The POETIC study assesses the benefit of peri-operative non-steroidal AI therapy using anastrozole or letrozole. Exemestane is excluded not for efficacy issues, rather because its steroidal structure may confound biological studies (13, 14).

3.1 Consultation on trial procedures and conduct

The perioperative setting of this study, together with the incorporation of primary biological endpoints, represents a new direction for UK breast cancer research, and will require changes in standard practice within many centres. We have therefore consulted widely with both

clinical colleagues and consumers to minimise changes to routine clinical practice, and ensure that the procedures and their timing are acceptable and practical.

Consultation with clinical colleagues

A focus group of interested clinicians and nurses met in October 2007 to identify any potential obstacles to the running of the study. This group highlighted the requirement for tissue taken from patients prior to their entry into POETIC to be stored within an HTA licensed laboratory. As a result of this meeting Pathway B (see Section 8) was introduced to accommodate patients diagnosed prior to having the opportunity to consider the trial. This group also identified ways of improving the flowcharts showing the patient pathways [Appendix 2].

Further to discussions in June 2008 at meetings of the UK Breast Intergroup and the NCRI's Breast Clinical Studies Group, a more pragmatic definition of essential tissue for this study has been developed, in order that only minimal changes to local standard practices and procedures are required to facilitate participation.

Consultation with consumers

Issues pertinent to consumers were presented to the NCRI consumer liaison group on 25 January 2007, and the patient information sheet reflects comments made at that meeting. Consumers were asked to consider whether research tissue should be taken from patients at the same time as diagnostic tissue, or taken after a diagnosis of ER positive breast cancer is confirmed. They were made aware that research tissue taken at diagnosis may need to be taken from patients who subsequently turn out to be ineligible for trial entry, and that taking tissue at that time allowed little time for patients to consider whether they wanted to donate tissue. The consumers preferred extra tissue for research at diagnosis to be taken at the same time as the routine core biopsy, and not after diagnosis is confirmed. Consumers also preferred any tissue taken to be used for research wherever possible.

Views of the North Trent Consumer Research Panel have been sought on the information sheet for donation of tissue at diagnosis.

The POETIC patient information sheets and consent forms, with earlier consumer comments incorporated, were reviewed by the Surrey, West Sussex and Hampshire Cancer Research Network Cancer Partnership Research Group. This group was also asked to consider the appropriate timeframes for patients to consider trial entry. Its view was that the diagnosis should be known before the trial is offered, and length of time available to consider trial entry was of secondary importance.

4. Trial Objectives

1. To determine whether perioperative endocrine therapy with an AI followed by standard adjuvant therapy improves outcome compared with standard adjuvant therapy alone in postmenopausal women with hormone receptor positive breast cancer.
2. To determine whether the proliferation marker Ki67 as measured by immunohistochemistry (IHC) in the excised cancer around 2 weeks after starting AI therapy will predict for time to recurrence (TTR) in the individual patient more effectively than the pre-treatment Ki67 value.

3. To determine whether molecular profiling 2 weeks after starting endocrine therapy predicts for long-term outcome in postmenopausal women with hormone receptor positive breast cancer better than at diagnosis.

4.1 Exploratory Objectives:

1. To deconstruct the underlying mechanisms that might explain the development of metastatic disease by correlating the genomic data of primary and metastatic tissue a) with clinical data of patients and b) against Ki67 and other molecular features measured at baseline and 2-week post-treatment.
2. To determine whether the amount of ctDNA in the plasma collected could act as measure of residual disease after surgery and predictor of relapse.

5. Study Design

For all patients who consent to enter POETIC, surgery should be booked prior to randomisation.

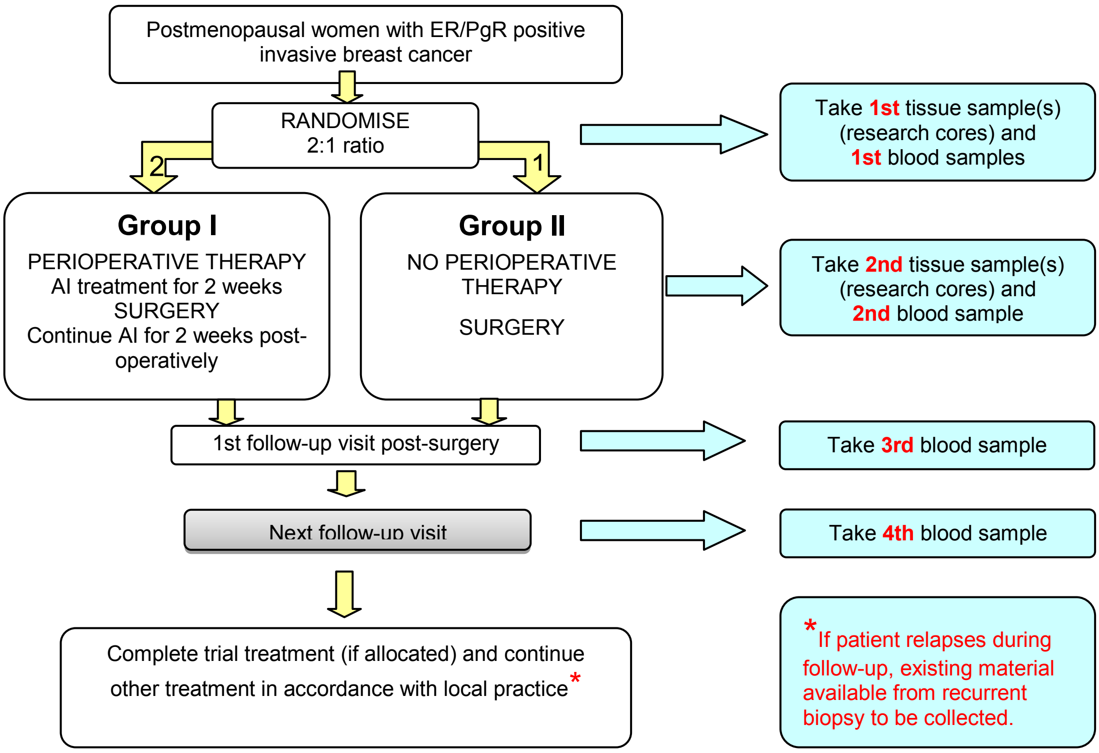

Choice of AI (anastrozole or letrozole) is to be declared by each participating centre at the outset of the trial.

For the purposes of national cancer waiting times, allocation to AI or no AI (following randomisation into POETIC) is classed as first treatment.

In order to maintain the scientific integrity of the trial it is essential that all non trial treatments are not influenced by the patient's allocation in POETIC (e.g. need to ensure that use of adjuvant AIs or chemotherapy does not differ between the two groups). In order to achieve this all non trial treatment should be given according to standard local policy.

6. Endpoints

6.1 Primary endpoints:

- Time to recurrence

6.2 Secondary endpoints:

- Relapse free survival
- Time to local recurrence;
- Time to distant recurrence;
- Overall survival;
- Breast cancer free survival;
- Proliferation rate (Ki67) at baseline core biopsy, and at surgical excision and
- Gene expression profile at core biopsy and at surgical excision.

6.3 Exploratory Endpoints

- Gene expression profile at metastatic excision;
- Mutation profile at core biopsy, surgical and metastatic excision and
- Somatic DNA alterations in the ctDNA that might be defined as coming from the primary tumour.

7. Patient Selection and Eligibility

7.1 Source of patients

Postmenopausal women with ER and/or PgR positive primary breast cancer will be recruited from breast cancer clinics within participating UK centres.

Hormone receptor positive breast cancer is defined as an Allred score of 3 or more, *or* a Histoscore of 2 or more, *or* more than 1% of positive cells for oestrogen receptor or progesterone receptor.

7.2 Number of patients

Approximately 4350 patients will be required plus the number required to complete POETIC sub-studies.

7.3 Inclusion criteria:

1) Post-menopausal women with core biopsy-proven hormone receptor positive invasive breast cancer. Postmenopausal is defined as a woman aged ≥50 years fulfilling any one of the following criteria:
   i) with amenorrhoea >12 months and an intact uterus;
   ii) has undergone a bilateral oophorectomy;
   iii) in women who have undergone a hysterectomy, then FSH levels within the postmenopausal range (utilising ranges from the testing laboratory facility) are required if the patient is aged <55 years; or
   iv) in women who have been on HRT within the last 12 months and therefore not amenorrhoeic, FSH levels within the postmenopausal range (utilising ranges from the testing laboratory facility) are required if the patient is aged <55 years.

2) No evidence of metastatic spread by standard assessment according to local guidelines
3) Standard adjuvant endocrine therapy indicated
4) A palpable tumour of any size , or a tumour with an ultrasound size of at least 1.5cm
5) WHO performance status of 0 or 1
6) Written informed consent to participate in the trial and to donation of tissue (fresh tissue and surplus tissue from diagnostic procedures) and blood samples.

7.4 Exclusion criteria:

1) Locally advanced/inoperable breast cancer
2) Evidence of metastatic disease
3) Previous invasive breast cancer (surgically treated DCIS or LCIS allowed)
4) Current bilateral breast cancer
5) Multiple unilateral tumours with different ER/PgR/HER2 status, grade or type (e.g. ductal vs lobular) i.e. anything that suggests two or more different cancers. Multifocal disease with homogenous ER/PgR/HER2 status, grade and type is allowed if at least one lesion is palpable or at least 1.5cm on ultrasound; the largest lesion should be used for sample collection and CRF completion.
6) Concurrent use (defined as use within 4 weeks prior to diagnostic tissue sample being taken) of HRT or any other oestrogen-containing medication (including vaginal oestrogens)
7) Previous use of oestrogen implants at ANY time
8) Prior endocrine therapy or chemotherapy for breast cancer
9) Any invasive malignancy diagnosed within previous 5 years (other than basal cell carcinoma or cervical carcinoma in situ)
10) Any severe co-incident medical disease, inability to give informed consent or unavailability for follow-up
11) Treatment with an unlicensed or investigational drug within 4 weeks before randomisation
12) Current, continuous, long term systemic steroid usage

8. Procedure for Obtaining Consent for Trial Entry & Tissue Donation

Selected centres will be designated as Biological Centres. Centres in England Wales and Northern Ireland will adhere to guidance from the Human Tissue Authority on taking and storing tissue for patients prior to their entry into POETIC, details of which are available from the POETIC trials office. Biological centres will provide samples in RNA-later® and one paraffin block for all consenting patients, taken prior to study entry and at surgery. Biological centres may enter patients into the study via Pathway A or Pathway B. They may also enter patients as 'non-biological' where individual circumstances make Pathways A and B impractical.

Pathway A: requires tissue to be taken from patients at the same time as the diagnostic core biopsy. Generic consent for research core biopsy samples should be gained prior to diagnostic core biopsy. The patient should be offered the trial as soon as hormone receptor positive breast cancer and eligibility are confirmed.

Pathway B: requires patients to be asked to donate additional research core biopsy samples taken after written informed consent and before randomisation.

All other centres (i.e. non-biological centres) are not required to provide samples in RNA-later®. Paraffin embedded tissue must be available from diagnostic tissue already taken.

Flowcharts showing the details for both biological and non-biological centres are provided in Appendix 2. Details of samples required for all pathways are provided in Appendix 3

All patients must have their excisional surgery booked prior to randomisation, and it is recommended that patients considered for POETIC are booked for their excisional surgery at the time of first referral.

Biological centres

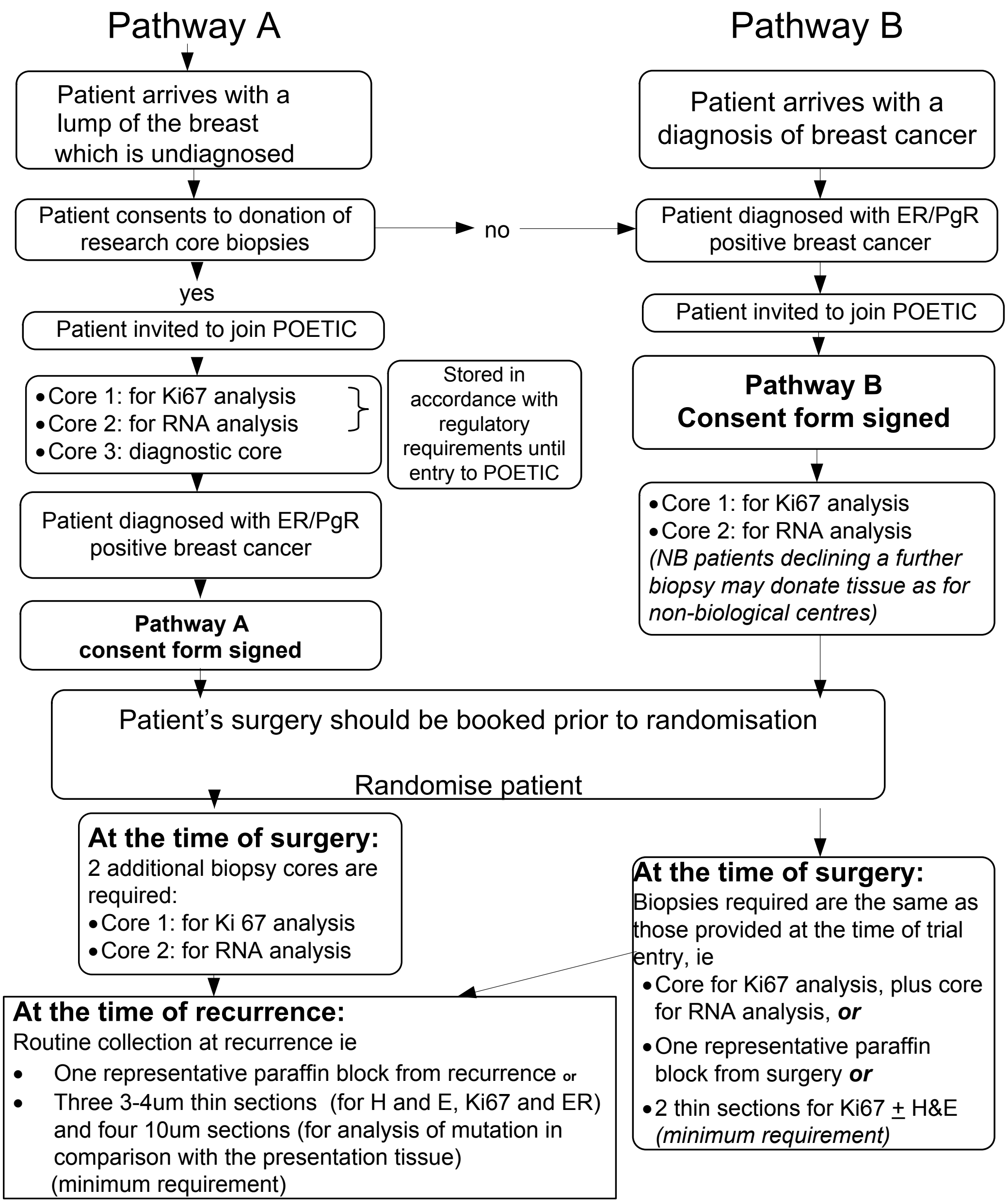

All other centres (non-biological centres)

Patient diagnosed with ER/PgR positive breast cancer

↓

Patient invited to join POETIC

↓

Consent form for non-biological centres signed

↓

- One representative paraffin block from diagnosis ***or***
- Ten 3-4µm sections + four 10µm sections ± H&E
- Two 3-4µm sections for Ki67 ± H&E *(minimum requirement)*

↓

Patient's surgery should be booked prior to randomisation

Randomise patient

↓

At the time of surgery
Tissue required is in the same form as that provided at trial entry, ie

- One representative paraffin block from surgery ***or***
- Two 3-4µm sections for Ki67 ± H&E *(minimum requirement)*

↓

At the time of recurrence:
Routine collection at recurrence, ie

- One representative paraffin block from recurrence **or**
- Three 3-4um thin sections (for H and E, Ki67 and ER) and four 10um sections (for analysis of mutation in comparison with the presentation tissue). (minimum requirement)

9. Biological Specimen Collection

9.1 Specimen Collection

Tissue Collection

The collection of biological samples is an essential part of this study. The minimum requirement is for paraffin embedded tissue to be provided for each patient both prior to trial entry and at surgery. An additional (optional) paraffin embedded tissue sample or sections from routine excision at the time of recurrence, are also requested. Options for the provision of this material are given in the diagrams above and in further detail in Appendix 3.

Centres are encouraged to provide additional material in tubes containing RNA-later® for additional biological studies. Those that provide this material are designated as Biological Centres. As well as paraffin embedded material, Biological Centres will provide one fresh sample in a tube containing RNA-later® (will be provided), taken before randomisation (at diagnosis for Pathway A; at trial entry for Pathway B), for all consenting patients whose tumour size permits multiple biopsies, Biological Centres will also provide a further fresh tissue sample in RNA-later® taken at surgery.

TRICC funding will facilitate the collection of fresh material for the secondary biological analyses on RNA, however subsequent scientific studies will only be undertaken once the scientific procedures have been agreed with the TMG and additional grant funding has been secured.

Blood Sample Collection

The following blood samples are required for all consenting patients:

I. **At randomisation**: two samples;1 x 5ml EDTA, 1 x 8.5ml PAXgene tube
II. **At surgery:** one 5ml sample in EDTA
III. **At first follow-up after surgery**: one 5ml EDTA sample
IV. **Late follow-up visit:** two samples; 1 x 5ml EDTA, 1 x 10ml Streck tube (not required if patient has relapsed)

Blood kits required for the study will be provided, and all centres should participate in the collection of blood samples.

Further details on storage and transfer of blood and tissue samples are given in Appendix 3, and full details of blood and tissue sampling collection and storage are given in trial specific Trial Guidance Notes, which should be followed for all samples taken in relation to the POETIC trial.

9.2 Use of tissue sample collection beyond POETIC

It is envisaged that all tissue taken at diagnosis from patients who do not ultimately enter POETIC is made available for other high quality ethics approved research. To this end, information offered to patients at the time of donating this tissue should make it clear that donated tissue may be made available to researchers.

10. Randomisation Procedure

Patients agreeing to enter the trial will have surgery booked prior to randomisation and scheduled for around two weeks ahead.

HRT users must stop HRT a minimum of four weeks before the baseline diagnostic biopsy is taken (HRT may affect tumour proliferation and hence the Ki67).

For both pathways A and B: patients should ideally be randomised around 14 days before their scheduled surgery date. Patients will be randomised into 2 groups in a 2:1 ratio as follows:

- PERIOPERATIVE THERAPY with AI and excisional surgery 14 days later (or a minimum of 10 days later);

or

- NO PERIOPERATIVE THERAPY with surgery at around 14 days later (or a minimum of 10 days later)

An eligibility checklist and randomisation checklist should be completed prior to randomisation. To randomise a patient, telephone ICR-CTSU on the dedicated randomisation line (see

below). The person randomising the patient will be asked to confirm that an eligibility checklist has been completed and to verify that the patient has signed the POETIC consent form (a sub-set of these will be the subject of a later audit). The person will also be asked for all the information on the randomisation checklist. A trial number and treatment allocation will be given over the telephone and later confirmed in writing.

The eligibility and randomisation checklists should be completed prior to randomisation.

To randomise a patient please telephone:

+44 (0)20 8643 7150 (Monday – Friday 09:00 – 17:00)

11. Trial Treatment

Allocation of treatment

Perioperative endocrine therapy, if allocated, should be started as soon as possible after randomisation.

Irrespective of treatment allocation, patients should not take any concomitant oestrogen-containing medication.

Choice of Investigational Medicinal Product (IMP)

PERIOPERATIVE THERAPY will be either
anastrozole 1mg/day
or letrozole 2.5 mg/day

Anastrozole is used outside its current licensed indication and letrozole is used within its licensed indication. Both are oral preparations formulated as tablets.

Participating centres should declare prior to enrolling patients whether they propose to use anastrozole or letrozole. Centres may, if they wish, change their practice to the alternative AI during the course of the trial, but should do so for all future patients. Before a change is made, the date of changing to an alternative AI should be agreed with ICR-CTSU.

Duration of treatment

Treatment should commence immediately after randomisation allowing duration of treatment before surgery to be as close as possible to 14 days. Ideally, patients should commence trial treatment exactly 14 days before surgery. Where necessary, a minimum of 10 days treatment before surgery is allowed in order to avoid delays to surgery. If surgery is delayed, the pre-treatment duration should be extended, and treatment continued at this daily dose without interruption until 14 days after the date of surgery, including the day of surgery.

12. Drug supplies & labelling

Normal commercial supplies should be used.

Study drug should normally be dispensed by the hospital pharmacy. For anastrozole, patient identifiers and local investigator details should be added to the label in accordance with Annex 13 (*Volume 4, Good manufacturing practices, Annex 13, Manufacture of investigational medicinal products)* by the local pharmacy. Letrozole is being used within its licensed indication therefore it can be labelled in accordance with the requirements for a dispensed medicine. All IMPs should also be labelled ‘for clinical trial use only’. Labelling requirements do not apply for study drug dispensed by a community pharmacy, however full drug accountability is required as detailed in the Trial Guidance Notes.

13. Non Trial Treatment

It is important to ensure that use of non-trial therapy, including consideration of participation in an adjuvant treatment trial, is not influenced by the patients’ treatment allocation within POETIC. If such a practice occurred, with differential use of adjuvant therapy between the groups, this would undermine the scientific integrity of the trial and affect its ability to reach its stated objectives. In order to avoid this, all non-trial therapy should be given according to standard local practice guidelines. All Non Trial Treatment, as described below, must be recorded in the Case Report Forms (CRF). CRF completion guidelines are contained in the Trial Guidance Notes.

13.1 Surgical Treatment

Patients may undergo either breast conserving surgery or mastectomy in accordance with local protocols and patient choice. Patients entered into this study must have subsequent confirmation of axillary node status. The axilla should be staged by axillary sampling, sentinel node biopsy (SNB) or axillary clearance. If sampling or SNB identifies axillary node involvement, axillary nodes should be resected or axillary radiotherapy undertaken in accordance with local protocols. In patients treated by breast conservation, clear margins should be achieved. Further re-excision to achieve a margin of clearance should be in accordance with local protocols. Where there is a close or involved deep margin following mastectomy, or axillary lymph node involvement, chest wall radiotherapy may be administered in accordance with local protocols (see below).

Primary breast reconstruction and other oncoplastic procedures to improve cosmetic outcome are acceptable in the POETIC trial.

13.2 Radiotherapy

Radiotherapy should be given, if required, after chemotherapy or surgery in keeping with local practice.

13.3 Adjuvant Endocrine therapy

All patients will be treated in accordance with local policy based on the national clinical guidelines that prevail at the time. It is expected that these patients will receive adjuvant endocrine therapy with either tamoxifen or an aromatase inhibitor, or each sequentially, for a

minimum of 5 years. It is recognised that choice and duration of endocrine therapy is a changing field and national policies may change during the course of the trial. Centres must declare their current policy prior to participation, and advise ICR-CTSU of any changes to that policy as they occur. For patients randomised to perioperative endocrine therapy continued treatment with the same AI in the adjuvant setting is not a study requirement.

13.4 Adjuvant Chemotherapy

Chemotherapy and supporting medications (including GCSF) may be offered in accordance with local policy. It is recommended that MDTs give due consideration to other factors, including grade at diagnostic core, when considering adjuvant chemotherapy for patients who have received perioperative AI treatment. This is because grade at surgery following two weeks of perioperative AI treatment may not be as reliable an indicator as it might otherwise have been.

13.5 Adjuvant Herceptin and Bisphosphonates

Adjuvant Herceptin should be given in accordance with national clinical guidelines.

At the time of preparing the POETIC protocol, there are no standard guidelines for adjuvant bisphosphonates; however evidence may emerge during the course of the trial to justify their use. Should such evidence emerge, adjuvant bisphosphonates should be given, if required, in accordance with agreed national guidelines.

14. Trial Evaluations

14.1 Staging investigations

Required staging investigations are according to local practice and thus are in keeping with standard UK practice in breast cancer management. No additional staging investigations are required.

14.2 Follow–up Investigations

Patients should be followed-up as per local practice for patients with early breast cancer who have been entered into a clinical trial. Follow-up data will be collected on annual follow-up CRFs, local/distant relapse CRFs, secondary cancer CRFs and death CRFs. Imaging and biochemical investigations will be carried out as clinically indicated where recurrence or other significant clinical problems are suspected.

To enable the 4th blood sample (late follow up) to be collected patients should be invited to attend clinic to consent and collect this sample.

15. Pharmacovigilance

15.1 Definitions

15.1.1 Serious Adverse Events (SAEs)

An SAE is any untoward medical occurrence that occurs after the commencement of randomised treatment and within 30 days of the last administration of the trial drug and:

- Results in death;
- Is life-threatening: *refers to an event in which the patient was at risk of death at the time of the event. It also refers to an event that would result in death with the*

continued use of the product; it does not refer to an event which hypothetically might have caused death if it were more severe;

- Requires inpatient hospitalisation or prolongation of existing hospitalisation: *admission to hospital overnight or prolongation of a stay in hospital was necessary as a result of the AE. Outpatient treatment in an emergency room is not itself an SAE, although the reasons for it may be. Hospital admissions/surgical procedures planned for a pre-existing condition before a patient is randomised to the study are not considered SAEs, unless the illness/disease deteriorates in an unexpected way during the study*;
- Results in persistent or significant disability/incapacity: *results in a significant or persistent change, impairment, damage or disruption in the patient's body function/structure, physical activities or quality of life;*
- Is a congenital anomaly/birth defect; or
- Any untoward medical occurrence requiring medical intervention to prevent permanent impairment or damage.

15.1.2 Serious Adverse Reactions (SAR)

A SAR is an SAE that has a definite, probable or possible causal relationship to the trial drug.

15.1.3 Suspected Unexpected Serious Adverse Reactions (SUSARs)

Any adverse reactions that have a suspected relationship to an IMP that are both serious and unexpected, as judged by the CI.

15.2 Causality (relatedness)

The assignment of causality for serious adverse events should be made by the investigator responsible for the care of the patient using the definitions in Table 1. If any doubt about the causality the investigator should inform ICR-CTSU who will notify the Chief Investigator. Pharmaceutical companies and/or other clinicians may be asked to advise.

Table 1 – Definitions for causality

Relationship	Description
**Unrelated**	There is no evidence of any causal relationship with the trial drug
**Unlikely**	There is little evidence to suggest there is a causal relationship (e.g. the event did not occur within a reasonable time after administration of the trial medication). There is another reasonable explanation for the event (e.g. the patient's clinical condition, other concomitant treatment)
**Possible**	There is some evidence to suggest a causal relationship (e.g. because the event occurs within a reasonable time after administration of the trial medication). However, the influence of other factors may have contributed to the event (e.g. the patient's clinical condition, other concomitant treatments)
**Probable**	There is evidence to suggest a causal relationship, and the influence of

	other factors is unlikely
**Definitely**	There is clear evidence to suggest a causal relationship, and other possible contributing factors can be ruled out
**Not assessable**	There is insufficient or incomplete evidence to make a clinical judgement of the causal relationship

15.3 Reporting of Serious Adverse Events/Reactions to ICR-CTSU

SAEs in this study that are fatal or life threatening or result in persistent or significant disability/incapacity should be reported using a Serious Adverse Event (SAE) form. Other SAEs that are definitely, probably, or possibly related to the randomised treatment (including prolongation of existing hospitalisation) should be reported using a Serious Adverse Reaction (SAR) form. SAEs that are hospitalisation or prolonged hospitalisation and unrelated or unlikely to be related to the study drug do not require reporting.

Medical and scientific judgement should be exercised in deciding whether reporting is appropriate in other situations, such as important medical events that may not be immediately life threatening or result in death or result in persistent or significant disability/incapacity.

Data on adverse events that are not serious in accordance with the above definition will not be collected.

15.4 Recording and Reporting of Serious Adverse Events

Serious adverse events, whether on an SAE or a SAR form, require immediate reporting by fax to ICR-CTSU within 24 hours of the Principal Investigator or designated representative becoming aware of the event.

Please fax SAE and SAR forms for the attention of POETIC Trial Manager to the ICR-CTSU Safety Desk

Fax: +44 (0)20 8722 4368 (Monday – Friday 09:00 – 17:00)

Forms must be completed, signed and dated by the Principal Investigator or designated representative.

15.5 Review of Serious Adverse Event

Events reported using an SAE form will be reviewed immediately by the Chief Investigator (or designated representative) for causality and expectedness.

Centres should respond as soon as possible to requests from the CI or designated representative (via ICR-CTSU) for further information that may be required for final assessment.

15.6 Expedited Reporting of SUSARs

If an SAE is defined as a SUSAR and is fatal or life threatening, ICR-CTSU will report this to the MHRA, the Main REC, and to the Co-Sponsors within 7 days from the date of definition.

If an SAE is defined as a SUSAR and is not fatal or life threatening, ICR-CTSU will report this to the MHRA and Main REC and to the Co-Sponsors within 15 days.

The Principal Investigator at all actively recruiting centres will be informed of any SUSARs occurring within the trial.

15.7 Follow-up of Serious Adverse Events

The patient must be followed-up until clinical recovery is complete and laboratory results have returned to normal, or until disease has stabilised. Information on final diagnosis and outcome of SAE which may not be available at the time the SAE was initially reported should be completed on the relevant part of the original SAE form within 15 days of resolution of the event and faxed to ICR-CTSU. This applies to all reported SAEs, whether reported on an SAE form or a SAR form.

15.8 Annual Reporting of Serious Adverse Reactions

An annual report will be provided to the MHRA and the Main REC at the end of the reporting year. This will be defined as the anniversary of the date when the Clinical Trials Authorisation (CTA) was obtained. This will include all related events reported on SAE and SAR forms, and a report from the Independent Data Monitoring Committee (IDMC).

Flow diagram for SAE reporting, and action following report

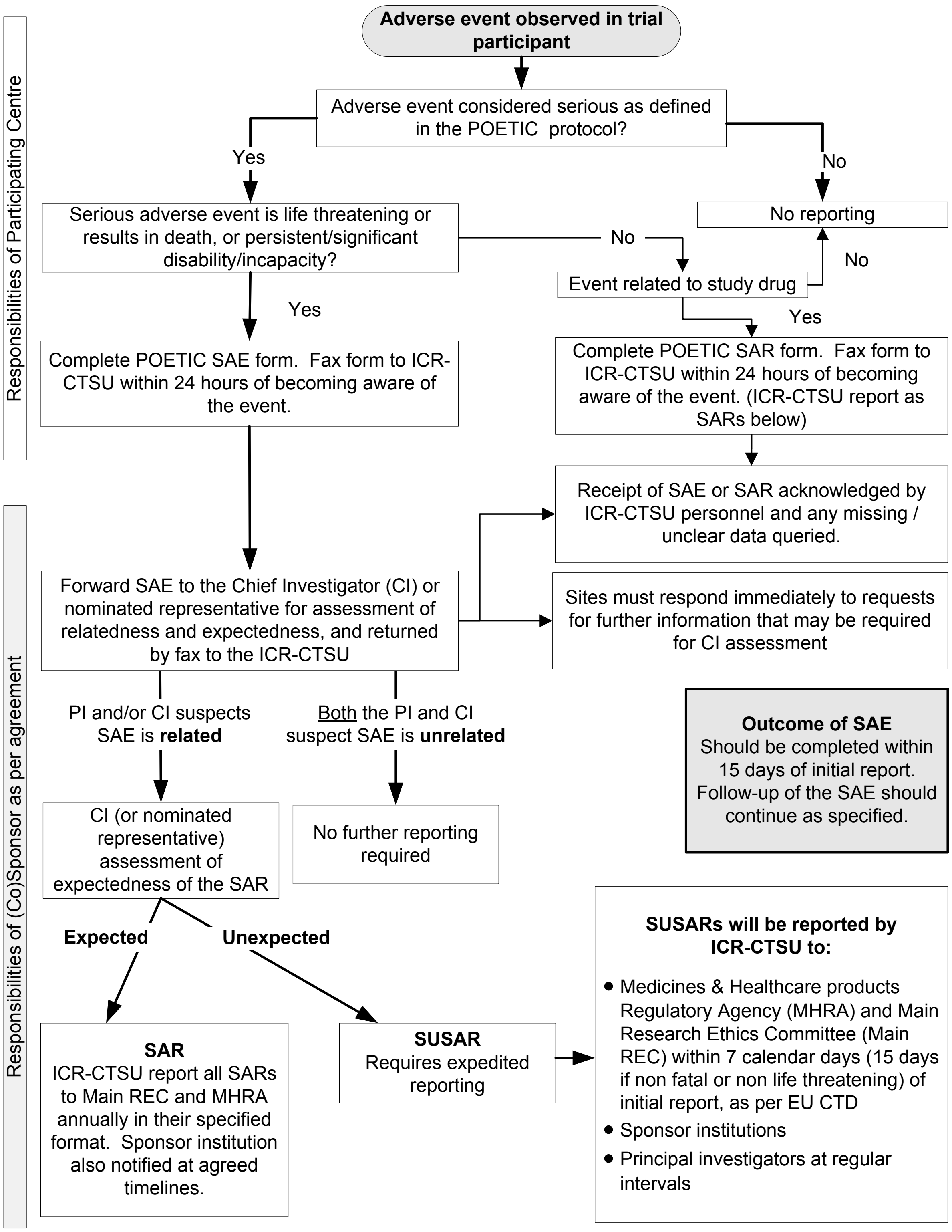

16. Statistical Considerations

16.1 Trial Hypothesis

POETIC is a randomised phase III clinical trial addressing the hypotheses:

- that 4 weeks perioperative treatment with an aromatase inhibitor will improve the time to recurrence in women with ER &/or PgR positive breast cancer compared with standard adjuvant treatment; and
- that the proliferation marker Ki67 following 2 weeks of aromatase inhibitor therapy can predict for long-term outcome in postmenopausal women with hormone receptor positive breast cancer better than at diagnosis.

16.2 Stratification

Randomisation will be stratified by:

- Participating Centre

Stratification by nodal status or grade is not possible as this will not be known at the time of randomisation. With approximately 4350 patients recruited, imbalances in prognostic factors between the treatment groups are unlikely.

Prognostic information on number of clinically involved nodes, Ki67, ER status, PgR status, HER2 status, clinical tumour size and grade will be recorded and may be used to adjust analyses.

16.3 Randomisation

Patients will be allocated in a 2:1 ratio (Perioperative therapy : No perioperative therapy) and randomisation will be conducted according to variable sized permuted blocks. A 2:1 allocation maximises information about perioperative effects of aromatase inhibitors whilst not compromising statistical power. By allocating more patients to the treatment group more information on the individual biological perioperative effects of these drugs will be available, in particular with respect to assessing how these effects reflect long term outcome.

16.4 Sample Size

The number of first relapses observed in this trial determines its ability to detect differences in outcome and defines the trial size. The relapse rate is expected to be low (8-10%), hence with approximately 4350 patients we expect to observe 320-400 first relapses - during the first 5 years of follow-up. As there are two primary aims the effect on each endpoint of this sample size is detailed below:

i) Time to Recurrence
If the true five year relapse rate in patients treated with immediate surgery is 10% then with 4350 patients it will be possible to detect a 3% improvement to 7% with 91% power (two sided alpha of 5%).

In order to allow for underestimation of the relapse rate potentially due to patients dying from other causes prior to breast cancer relapse, the original sample size of 4,000 patients has

been increased by a further 350 patients (taking the total sample size to approximately 4,350 patients, plus the number required to complete the POETIC sub-studies).

ii) Comparison of the prognostic value of Ki67 at surgery and baseline in the perioperative therapy group:

It will be possible to detect a 1.3 fold difference in the ability of Ki67 to predict time to recurrence, e.g. from 1.5 at baseline to 2.0 (1.5 x 1.3) or more at surgery (90% power, two sided 5% significance level). However, this will be an over-estimate of the detectable difference because 2 week and baseline Ki67 are correlated (R squared 30%), hence there would be expected to be some correlation between their ability to predict recurrence.

In addition to the primary endpoints, consideration has been given to the ability to identify molecular signatures as prognostic factors for outcome. No formal sample size considerations will be supplied to address this question. However, identifying molecular signatures requires a large amount of data. Paik et al (14), for example, used data from 447 patients to select a panel of 21 genes from a set of 250 candidate genes; this panel was then tested in a series of 668 patients. There are good grounds for recommending that the current trial employs more than 1100 patients to address this question because the molecular signature will also incorporate gene expression changes. Paik et al also found that poor grade (versus good or intermediate grade) was a significant independent prognostic factor in multivariate analysis including their recurrence score. We will be able to test the effect of both grade and Ki67 as independent factors to molecular signatures determined in this study.

16.5 Analysis Plan

Time to Recurrence (TTR) is defined as time from randomisation to local, regional, or distant tumour recurrence or death from breast cancer without prior notification of relapse. Second primary cancers and intercurrent deaths will be treated as censoring events. Patients who are alive and disease free will be censored at the date last seen alive.

Intention to treat comparisons will be tested as above with and without adjustment for centre, choice of aromatase inhibitor and known prognostic factors.

Overall survival will be measured from date of randomisation to date of death from any cause.

A sensitivity analysis endpoint will look at breast cancer free survival and relapse free survival (i.e. local, regional or distant tumour recurrence, or breast cancer death). Second primary cancers and deaths from non-breast cancer causes in the absence of breast cancer relapse will be treated as censoring events.

Analyses will be based on the intention to treat principle. For the comparison between perioperative versus no perioperative treatment the principal analysis will be a logrank comparison. Comparison of the predictive value of Ki67 at surgery in the perioperative therapy and non perioperative therapy groups will be undertaken using Cox regression, comparing the estimates of the hazard ratios obtained in each treatment group. Sample size considerations have been based on a linear relationship between the log hazard ratio and log Ki67 but as an exploratory analysis the optimum relationship will be obtained by fitting centred linear, quadratic and cubic relationships (on three degrees of freedom). Similar methods will be used

for the comparison of the predictive value of Ki67 at surgery and baseline in the AI comparison between the two timepoints will then involve comparing the change in the log-likelihood.

Cox regression methods will also be used for multivariate analyses (to further adjust for clinical factors likely to influence prognosis) and to estimate the hazard ratio and its associated confidence intervals and to test for interactions. The proportional hazards assumption will be checked to validate the use of Cox regression. The proportion of patients relapse free and surviving according to follow up time will be presented as Kaplan-Meier survival curves with fixed term survival estimates. Baseline characteristics will be described by randomised treatment group and correlations between baseline characteristics and biological markers will be investigated using Spearman Rank correlation or a chi-squared test. Comparisons will be performed using simple parametric, exact, non-parametric or chi-squared tests as appropriate. Tests will be two-sided and 95% confidence intervals will be used.

Data on compliance with treatment allocation and actual duration of hospital inpatient stay for surgery will be collected and compared between the groups.

16.6 Interim Analyses and role of Independent Data Monitoring Committee (IDMC)

An IDMC will be instigated to monitor the progress of the trial. It will meet in confidence at regular intervals, and at least annually. A report of the findings and recommendations will be produced following each meeting. This report will be submitted to the Trial Management Group and Trial Steering Committee, the main REC and the MHRA, as required.

Interim review of side effects, time to recurrence, relapse free and overall survival for all randomised patients will be performed at approximately yearly intervals. These analyses will be supplied in strict confidence by the trial statistician to the IDMC together with any other analyses that the IDMC may request. No results on survival or time to recurrence or biomarkers will be made available to investigators or any other party until at least two years after the last patient is entered or sufficient events have been observed to allow primary analysis to be undertaken, unless the IDMC determines that it would be unethical to withhold the interim results.

The main criterion for early stopping of the trial by the Trial Steering Committee, upon advice from the IDMC and endorsement from the Trial Management Group, will be that evidence from the trial and from other sources suggests a) proof beyond reasonable doubt that for all, or for some types of patient, one treatment regimen is clearly indicated or contra-indicated in terms of a net difference in relapse free and/or overall survival or toxicity, and b) evidence exists that might reasonably be expected to influence routine clinical practice. Criterion for the above will usually be a difference in time to recurrence, relapse free and overall survival at any stage significant at $p < 0.001$ (Haybittle-Peto interim stopping rule) by overall log-rank analysis. Use of this criterion will not materially affect the overall alpha in the final analysis.

The IDMC will, however, reserve the right to release any data on outcome or side-effects through the Trial Steering Committee to the Trial Management Group (and if appropriate to

participants) if it determines at any stage that the combined evidence from this and other studies justifies it.

17. Study organisation

ICR-CTSU will be responsible for the collection of trial data, and for monitoring receipt and transfer of biological specimens. On receipt at ICR-CTSU, completed CRFs will be reviewed for data anomalies and their receipt recorded. Any data queries arising from initial review will be sent to the relevant centre for resolution. Following initial review, data items from the CRFs are entered centrally into the clinical study database at ICR-CTSU. Transfer of biological specimens will be monitored using a system of tracking forms sent by centres to inform ICR-CTSU of the movement of all biological samples. The Department of Academic Biochemistry at Royal Marsden Hospital will notify ICR-CTSU of all samples received. All data will be handled, computerised and stored in accordance with the Data Protection Act 1998.

17.1 Data monitoring plan

Data monitoring will primarily be conducted using central statistical monitoring, and any systematic inconsistencies identified may trigger monitoring visits to centres. Further monitoring visits may be conducted at the request of participating centres, or by random selection. Monitoring visits will be conducted at a random sub-set of participating centres and the extent and timing of this exercise will be determined by central statistical monitoring.

18. Follow-up management and completion of Case Report Forms (CRFs)

CRF completion guidelines are contained in the Trial Guidance Notes. The Trial Management Group reserves the right to amend or add to the CRFs as appropriate. Such changes do not constitute a protocol amendment, and revised or additional forms should be used by centres in accordance with the guidelines provided by ICR-CTSU.

18.1 Follow-up

Annual follow-up data will be collected for as long as the Trial Management Group consider it is contributing to the research question and will include as a minimum sites of recurrence, time of recurrence, mortality, and cause of death. Information on second primary breast cancers and other second primary tumours will also be recorded. Regular (annual or bi-annual) imaging of the breasts (e.g. mammography or MRI) should be part of the follow-up protocol according to local practice.

18.2 Relapse

The date of relapse is taken as the date of first confirmed recurrence by an appropriate investigation such as cytology, histology, or imaging wherever possible. In the absence of such confirmation, the date of first clinical suspicion will be taken provided that suspicion leads to a change or re-introduction of anti-cancer therapy. The management of recurrence will be at the discretion of the clinician. Follow-up information should continue to be provided until the patient dies.

19. Patient Protections and Ethical considerations

The study has been approved by London - South East Research Ethics Committee. Before entering patients, the Principal Investigator at each participating centre is responsible for gaining Site Specific Assessment and Research and Development approval.

Patients entering the study via Pathway A will be asked to sign *either* the generic consent form approved by London - South East Research Ethics Committee for use within POETIC, *or* a consent form approved by a Research Ethics Committee for the tissue bank holding tissue pending entry to POETIC, provided a copy of the information and blank consent form have been approved by the Chief Investigator. Prior to entering POETIC patients should be asked to sign the POETIC consent form after receiving both verbal and written information. This form incorporates consent to the biological studies. All consent forms must be countersigned by the Principal Investigator or a designated individual. A record of who the designated individuals are and the circumstances under which they may countersign consent forms must be clearly documented at the research site as part of the Delegation Responsibilities Log. This log, together with original copies of all signed patient consent forms, must be available for inspection.

The POETIC patient information sheet should be provided in addition to any standard patient information sheets for aromatase inhibitors that are provided by the centre and which are used in routine practice.

19.1 Liability/Indemnity/Insurance

Indemnity for participating hospitals is provided by the usual NHS indemnity arrangements.

19.2 Patient Confidentiality

The patient's full name, date of birth, hospital number and NHS number (CHI number in Scotland) will be collected at randomisation to allow tracing through GP and national records to assist with the collection of long term follow-up information. The personal data recorded on all documents will be regarded as confidential, and any information which would allow individual patients to be identified will not be released into the public domain.

The investigator must keep a separate log of patients' trial numbers, names, addresses and hospital numbers. The investigator must maintain trial documents, which are to be held at the participating centre (e.g. patients' written consent forms), in strict confidence. The investigator must ensure the patients' confidentiality is maintained.

ICR-CTSU will maintain the confidentiality of all patient data and will not reproduce or disclose any information by which patients could be identified. Representatives of ICR-CTSU and the regulatory authorities are required to have access to patient notes for quality assurance purposes. Patient confidentiality will be respected at all times. (In the case of special problems and/or government queries, it is also necessary to have access to the complete study records, provided that patient confidentiality is protected).

20. Withdrawal of patients from the trial treatment

20.1 Withdrawal of patients from trial treatment

Patients who do not receive their allocated treatment for any reason should be treated at the discretion of their clinician. Unless the patient requests otherwise, all CRFs, including long term follow-up, should be completed, regardless of treatment actually received, as analyses of all outcome efficacy data will be on the basis of intention to treat (i.e. all randomised patients). A trial deviation form should be completed to record details of deviation from treatment allocation. Patients are asked prior to randomisation to consent to follow-up should they withdraw from their allocated treatment (see patient information sheet and consent form), and any patient unwilling to give that assurance prior to trial entry should not be randomised. Patients are, however, free to reverse that decision at any time without giving a reason (see below).

20.2 Withdrawal of patients from trial follow-up

A trial deviation form should be completed in the unlikely event that the patient withdraws consent for further follow-up data to be collected. If this situation is suspected, clarification should be sought to ensure that the patient is not simply withdrawing from allocated treatment (as above). In the extremely unlikely event that the patient wishes to have their data removed from the trial completely (the implications of this should be discussed with the patient to ensure that this is their intent) this should be indicated as such on the trial deviation form.

21. Completion of the study and definition of study end date

The study end date is deemed to be the date of last data capture.

22. Research Governance

22.1 Trial Administration

The Co-sponsors of the POETIC trial are The Institute of Cancer Research (ICR) and the Royal Marsden NHS Foundation Trust (RMH), the Chief Investigator's host institution. Sponsorship activities and delegated responsibilities are shared between RMH and ICR, in accordance with The Medicines for Human Use (Clinical Trials) Regulations 2004 as amended and in line with the Research Governance Framework for Health and Social Care and the principles of GCP. Both parties agree to allow inspection of their premises by the competent authorities. Responsibilities of the Co-sponsors are set out in an agreement letter between ICR and RMH.

22.1.1 Trial Administration - Royal Marsden Hospital Responsibilities

RMH has sponsorship responsibility for obtaining authorisation and appropriate ethics committee opinion (Part 3 of the Regulations) and for pharmacovigilance (Part 5 of the Regulations).

The following responsibilities have been delegated:

The Chief Investigator:

- Selection of investigators

To the Chief Investigator or a named deputy delegated in his absence:

- Prompt decision as to which serious adverse reactions are SUSARs; and
- Prompt reporting of that decision to ICR-CTSU, Section of Clinical Trials, The Institute of Cancer Research for onward reporting to the licensing authority.

To The Institute of Cancer Research (through ICR-CTSU):

- Ensuring an appropriate ethics opinion has been sought, and any amendments have been approved;
- Giving notice of amendments to protocol, make representations about amendments to the main REC;
- Giving notice that the trial has ended;
- Keeping records of all serious adverse events (SAEs) reported by investigators;
- Ensuring recording and prompt reporting of serious adverse reactions (SARs) to the Chief Investigator;
- Reporting to the MHRA and main REC any suspected serious adverse reactions which the Chief Investigator considers to be unexpected (SUSARs);
- Ensuring Principal Investigators are informed of all SUSARs;
- Providing all SUSARs, including those in third countries, to the MHRA for inclusion on the European database; and
- Providing an annual list of SARs and a safety report to the licensing authority and Sponsors.

To participating centres:

- Ensuring recording and prompt reporting of SAEs/SARs to ICR-CTSU, Division of Clinical Studies, The Institute of Cancer Research.

22.1.2 Trial Administration - The Institute of Cancer Research Responsibilities

ICR has responsibility for ensuring the trial is conducted in accordance with the principals of Good Clinical Practice (Part 4 of the Regulations).

The following responsibilities have been delegated:

To RMH:

- Taking appropriate urgent safety measures – delegated to the Chief Investigator.

To participating centres:

- Putting and keeping in place arrangements to adhere the principles of GCP;
- Keeping a copy of all 'essential documents' (as defined under the principles of GCP) and ensuring appropriate archiving and destruction of documentation once the trial has ended as required by regulation 31 of the principal Regulations of the Medicines for Human Use (Clinical Trials) Regulations 2004 implementing the commission directive 2005/28EC;
- Ensuring investigational medicinal products (IMPs) are made available to subjects free of charge; and

- Taking appropriate urgent safety measures.

Responsibilities are defined in an agreement between an individual participating centre and the co-sponsors.

ICR is responsible for administering funding and co-ordinating any required legal agreements and investigator statements.

The delegation of sponsorship responsibilities does not impact on or alter standard NHS indemnity cover. The agreement of delegated responsibilities is viewed as a partnership and as such it is necessary to share pertinent information between ICR and RMH/Chief Investigator, including proposed inspections by the MHRA and/or other regulatory bodies.

22.2 Protocol Compliance & Initiation

The POETIC trial is being conducted in accordance with the professional and regulatory standards required for non-commercial research in the NHS under the EU Directive. Before activating the trial, participating centres are required to sign an agreement accepting responsibility for all trial activity which takes place within their centre.

Sites may commence recruitment once centre agreements have been signed by both parties, trial documentation is in place, and a telephone site initiation has taken place. Site initiation visits will be conducted at sites where the Principal Investigator has requested one, or where ICR-CTSU thinks that it is appropriate. Site initiation visits are not considered necessary for sites that have experience with other ICR-CTSU managed phase III breast cancer trials, or where staff have attended the Investigator Launch meeting.

22.3 Data Acquisition & On-Site Monitoring

On-site monitoring, or auditing, will be based on a risk-based strategy. ICR-CTSU staff may visit centres to confirm that agreements are being adhered to, specifically to carry out source data verification and confirm compliance with the protocol and the protection of patients' rights as detailed in the Declaration of Helsinki 1964 as amended October 1996. By participating in the POETIC trial, the Principal Investigators at each centre are confirming agreement with his/her local NHS Trust to ensure that:

- sufficient data is recorded for all participating patients to enable accurate linkage between hospital records and CRFs;
- source data and all trial related documentation are accurate, complete, maintained and accessible for monitoring and audit visits;
- all staff at their centre who are involved with the trial will meet the requirements of the EU Directive;
- original consent forms are dated and signed by both patient and investigator and are kept together in a central log together with a copy of the specific patient information sheet(s) given at the time of consent;
- copies of CRFs are retained for 15 years to comply with international regulations; and
- staff will comply with the protocol and Trial Guidance Notes for the POETIC trial.

ICR-CTSU will monitor receipt of CRFs and evaluate incoming CRFs for compliance with the protocol, inconsistent or missing data.

Participating centres may be monitored by ICR-CTSU and possibly by Health Authorities. Monitoring by ICR-CTSU aims to verify compliance with the protocol and to conduct source data verification (SDV).

Site monitoring will be conducted at a proportion of participating centres at least once during the course of the trial. If a monitoring visit is required ICR-CTSU will contact the centre to discuss dates of proposed visit. Once a date has been confirmed a list of patients whose notes will be monitored during the visit will be sent to the centre. This list will be sent out in advance to give sufficient time for the notes to be made available. The Trial Statistician will decide what percentage of patients is to be monitored.

If any problems are detected in the course of the monitoring visits then the Principal Investigator and ICR-CTSU will work together to resolve issues and, if necessary, to determine the centre's future participation in the study.

22.4 Archiving

Essential trial documents should be retained according to local policy and for a sufficient period for possible inspection by the regulatory authorities (at least 5 years after the date of last data capture). Documents should be securely stored and access restricted to authorised personnel.

22.5 Data Protection Act (DPA)

ICR-CTSU will comply with all aspects of the DPA 1998. Any requests from patients for access to data about them held at ICR-CTSU should be directed to the Trial Manager in the first instance, who will refer the request to the Data Protection Officer at The Institute of Cancer Research.

22.6 Financial Matters

The trial is investigator designed and led, and has been approved by Clinical Trials Advisory & Awards Committee (CTAAC) of Cancer Research UK, and meets the criteria for R&D support as outlined in the Statement of Partnership on Non-Commercial R&D in the NHS in England.

The trial has received funding from Cancer Research UK. If additional financial support is received from any other source this will be made apparent to the approving Main REC and CTAAC, but will not require a protocol amendment.

No individual per patient payment will be made to Trusts or investigators, but NCRN (or regional equivalent) network resources should be made available, as the trial is part of the NIHR portfolio.

22.7 Clinical risk assessment

Generic Risk Assessment Hazards to patients, study and organisation have been performed for the POETIC trial.

23. Publication policy

The main trial results will be published in the name of the trial in a peer-reviewed journal, on behalf of all collaborators. The manuscript will be prepared by a writing group, appointed from amongst the Trial Management Group, and high accruing clinicians. All participating centres and clinicians will be acknowledged in this publication together with staff from ICR-CTSU. All presentations and publications relating to the trial must be reviewed and approved by the Trial Management Group on whose behalf publications should usually be made. Authorship of any secondary publications, e.g. relating to the various biological studies, will reflect the intellectual and time input into these studies, and will not be the same as on the primary publication. No investigator may present or attempt to publish data relating to the POETIC trial without prior permission from the Trial Management Group.

24. References

1. Fisher B, Saffer EA, Rudock C, Coyle J, Gunduz N et al. Effect of local or systemic treatment prior to primary tumour removal on the production and response to a serum growth-stimulating factor in mice. *Cancer Res* 1989b; 49: 2002-2004.

2. Early Breast Cancer Trialists' Collaborative Group. Effects of chemotherapy and hormonal therapy for early breast cancer on recurrence and 15 year survival: an overview of the randomised trials. *Lancet* 2005; 365: 1687-1717.

3. Choy A. Mc Culloch P. Induction of tumour cell shedding into effluent venous blood breast cancer surgery. *Br J Cancer* 1996; 73 (1): 79-82.

4. Fisher B, Gunduz N, Coyle J, Rudock C, Saffer E. Presence of a Growth-stimulating Factor in Serum following Primary Tumour Removal in Mice. *Cancer Res* 1989a; 49: 1996-2001.

5. Dowsett M, Smith IE, Ebbs SR, Dixon JM, Skene A, Griffith C, Boeddinghaus I, Salter J, Detre S, Hills M et al on behalf of IMPACT Trialists. Short-Term Changes in Ki67 during Neoadjuvant Treatment of Primary Breast Cancer with Anastrozole or Tamoxifen Alone or Combined Correlate with Recurrence-Free Survival. *Clin Cancer Res* 2005; 11: 951s-958s.

6. Dowsett, M, Smith IE, Ebbs SR, Dixon JM, Skene A, Griffith C, Boeddinghaus I, Salter J, Detre S, Hills M et al. Proliferation and apoptosis as markers of benefit in neoadjuvant endocrine therapy of breast cancer. *Clin Cancer Res* 2006; 12: 1024s-1030s.

7. Dowsett M, Smith IE, Ebbs SR, Dixon JM, Skene A, A'Hern R, Salter J, Detre S, Hills M, Walsh G. Prognostic value of Ki67 expression after short-term presurgical endocrine therapy for primary breast cancer. *J Natl Cancer Inst* 2007; 99: 167-170.

8. Van de Vijver MJ, He YD, Van't Veer LJ, Dai H, Hart AA, Voskuil DW, Schreiber GJ, Peterse JL, Roberts C, Marton MJ et al. A Gene-expression signature as a predictor of survival in breast cancer. *N Eng J Med* 2002; 347: 1999 – 2009.

9. MINDACT Study UKCRN Portfolio Database Information. http://pfsearch.ukcrn.org.uk/StudyDetail.aspx?TopicID=1&StudyID=2516. *Accessed 30 January 2008.*

10. Paik S. Molecular profiling of breast cancer. *Curr Opin Obstet Gynecol* 2006; 18: 59-63.

11. Mackay A, Dixon JM, Urruticoechea A, Dexter T, Iravani M, Fenwick K, Young O, White S, Miller WR, Evans DB, Ashworth A, Dowsett M. Molecular determinants of aromatase inhibitor sensitivity in primary breast cancer. *Breast Cancer Res Treat* 2005; 94: S48 Abstr 1029.

12. Smith IE, Walsh G, Skene A, Llombart A, Mayordomo JI, Detre S, Salter J, Clark E, Magill P, Dowsett M. A phase II placebo-controlled trial of neoadjuvant anastrozole alone or with gefitinib in early breast cancer. *J Clin Oncol* 2007 25:3816-3822.

13. Spinelli R, Jannuzzo MG, Poggesi I, Frevola L, Broutin F, Cicioni P, Marrari P, LeCoz F. Pharmacokinetics (PK) of Aromasin (exemestane, EXE) after single and repeated doses in healthy postmenopausal volunteers (HPV). Eur J Cancer 1999; 35 (S4): S295 Abstr 1185.

14. Lonning P, Pfister C, Martoni A, Zamagni C. Pharmacokinetics of third generation aromatase inhibitors. *Semin Oncol* 2003; 30; (4 S14): 23-32.

15. Paik S, Shak S, Tang G, Kim C, Baker J, Cronin M, Baehner FL, Walker MG, Watson D Park T et al. A multigene assay to predict recurrence of tamoxifen-treated, node negative breast cancer. *N Engl J Med* 2004; 351: 2817-2826.

Appendix 1 Glossary of Terms

AI	Aromatase inhibitor
BASO	British Association of Surgical Oncology
ctDNA	circulating tumour DNA
CI	Chief investigator
CRF	Case Report Form
CTA	Clinical Trials Authorisation
CTAAC	Clinical Trials Advisory and Awards Committee
CXR	Chest X-Ray
DNA	DeoxyriboNucleic Acid
DPA	Data Protection Act
EBCTCG	Early Breast Cancer Clinical Trialists' Collaborative Group
ER	Oestrogen receptor
EU	European Union
FBC	Full Blood Count
FSH	Follicle Stimulating Hormone
GCP	Good Clinical Practice
GP	General Practitioner
HER2	Human Epidermal growth factor Receptor 2
HPV	Healthy postmenopausal volunteer
HRT	Hormone Replacement Therapy
HTA	Human Tissue Authority
ICR-CTSU	The Institute of Cancer Research Clinical Trials and Statistics Unit
IDMC	Independent Data Monitoring Committee
IHC	Immunohistochemistry
IMPs	Investigational Medicinal Products
ISRCTN	International Standard Randomised Controlled Trial Number
Main REC	Main Research Ethics Committee
MHRA	Medicines & Healthcare products Regulatory Agency
NCRI	National Cancer Research Institute
NCRN	National Cancer Research Network
NIHR	National Institute for Health Research
OS	Overall Survival
PK	Pharmacokinetics
PgR	Progesterone receptor
RFS	Relapse Free Survival
RMH	Royal Marsden NHS Foundation Trust
RNA	RiboNucleic Acids
SAE	Serious Adverse Event
SAR	Serious Adverse Reaction
SDV	Source Data Verification
SNB	Sentinel Node Biopsy
SUSAR	Suspected Unexpected Serious Adverse Reaction
TMG	Trial Management Group
TSC	Trial Steering Committee
WHO	World Health Organisation

Appendix 2 - Patient Pathways

POETIC Study
Patient Pathway A, and timelines for tissue sample collection
Identify postmenopausal patients with high likelihood of cancer

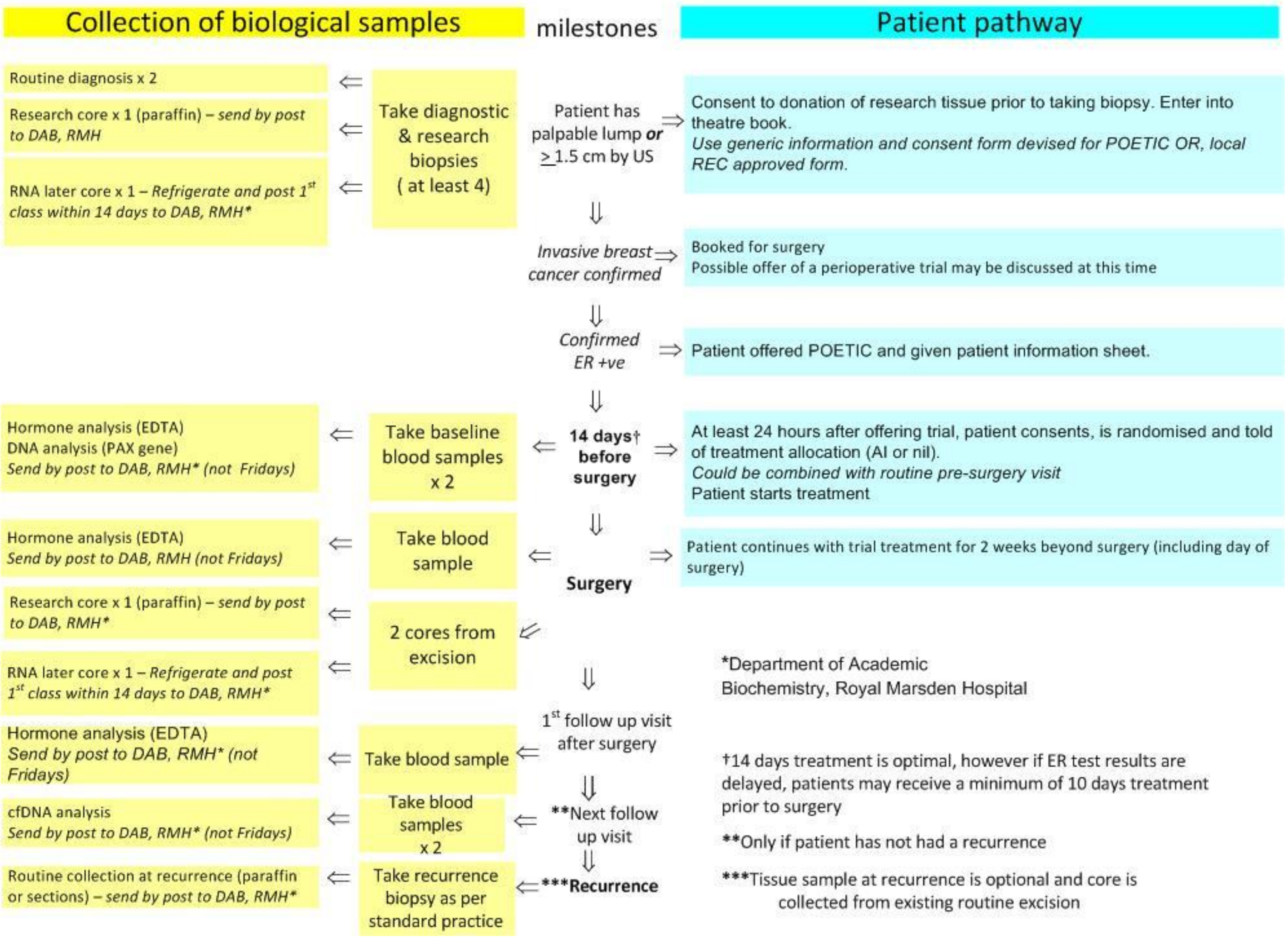

POETIC Study

Patient Pathway B, and timelines for tissue sample collection

Identify postmenopausal patients with invasive cancer confirmed

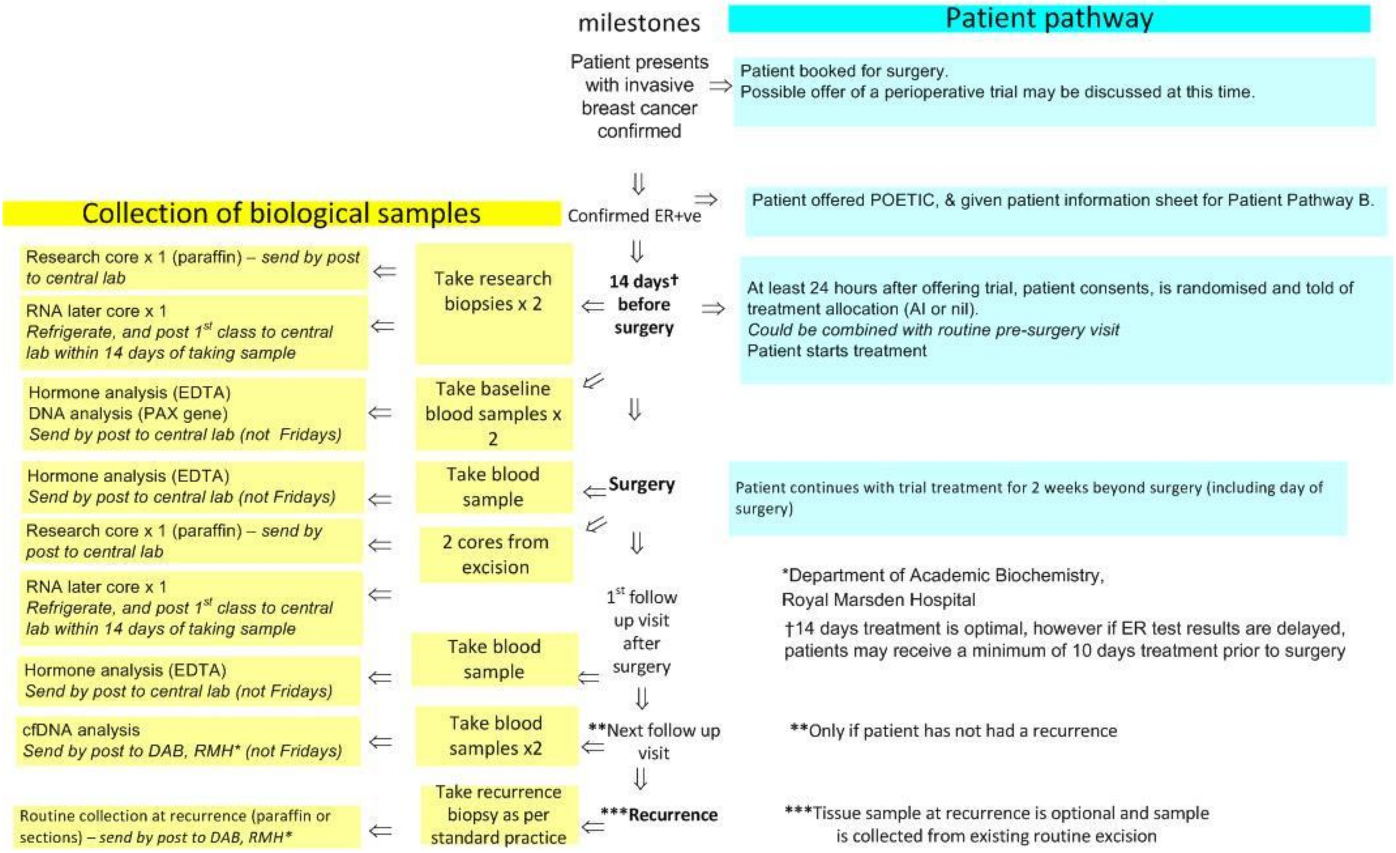

Appendix 3 – Tissue sample collection and analysis

A. Sample collection:

Tissue samples for patients entering POETIC from Biological Centres:

At diagnosis (pathway A) or immediately before randomisation (pathway B): 2 core biopsies (14-guage) are required ***in addition*** to those taken for diagnostic purposes. One is to be placed into formalin and the other placed in RNA-later® in tubes provided. All formalin fixed biopsies will be embedded in paraffin wax at the local histopathology department. If possible samples in RNA-later® should be refrigerated overnight at 4°C to allow thorough penetration of the tissue. If refrigeration is not available, samples can be stored at 25°C in RNAlater® Solution for up to 1 week without significant loss of RNA quality.

a. The sample in RNA-later® and the paraffin block, without prior sectioning, are to be sent by post to the Department of Academic Biochemistry, Royal Marsden Hospital either immediately the patient enters POETIC or together with samples taken at surgery (see below).

b. **At surgery:** 2 core biopsies (14-guage) to be taken immediately the tumour has been excised. One is to be placed into formalin and the other placed in RNA-later® in tubes provided. All formalin fixed biopsies will be embedded in paraffin wax at the local histopathology department. Samples in RNA-later® are to be stored as above, and both samples sent by post to the Department of Academic Biochemistry, Royal Marsden Hospital as soon as possible.

Kits for the collection of samples in RNA-later® are provided. Packaging, labelling and tracking is to be carried out in accordance with the Trial Guidance Notes.

Tissue samples from patients entering POETIC from all other centres

Tissue must be provided for all patients using one of the three options below. The option chosen should be on a case by case basis; however the type of tissue provided from diagnosis should be the same as that provided at surgery. The options for provision of tissue are:

a. **One tissue block of the diagnostic and excision samples**. This is the preferred option. From each of these, 10 thin (3 micron) section and 4 thick (10 micron) sections will be taken. Sections will be taken such as not to exhaust the blocks, which will be returned to the original pathology laboratory.

b. **Provision of the above sections on slides**, cut by the originating pathology laboratory

c. **2 thin (3 micron) sections.** This is the minimum for the patient to be eligible.

Tissue sample from all patients all pathways:

At the time of recurrence, an additional FFPE tissue block or sections, from routine excision at the time of recurrence, are optionally collected.

Blood samples – all centres
All centres must participate in the collection of blood samples (although individual patients may still enter POETIC and decline donation of blood samples):

a. **At randomisation:** 2 blood samples; one in EDTA and one in PAXgene (tubes provided).
b. **Up to 24 hours before surgery:** one blood sample in EDTA.
c. **At first follow-up visit after surgery:** one blood sample in EDTA.
d **Late follow-up visit:** 2 blood samples; one in EDTA and one in Streck (not required if patient has relapsed).

All blood samples are to be posted to the Department of Academic Biochemistry, Royal Marsden Hospital on the same day the sample is taken using the blood kits provided. Sample collection, packaging, labelling and tracking are to be carried out in accordance with the Trial Guidance Notes.

Patient consent procedures provided in the Trial Guidance Notes should be followed.

B. Sample analysis:

The formalin fixed core biopsies will be sectioned for the analysis of the proliferation marker, Ki67, by immunohistochemistry. Other sections will be stored after coating with paraffin wax for assessment of additional biomarkers that are candidates as being involved in response or resistance to hormonal treatment or in other aspects of breast cancer biology.

The core biopsies contained in RNA-later® will be analysed for RNA profiles and/or changes in the DNA of the tumour.

The EDTA-preserved blood samples will be centrifuged and the plasma stored frozen for the analysis of oestrogen levels. Residual plasma will be stored frozen for the analysis of biomarkers that may be related to the efficacy of treatment or the prognosis of the patient.

The blood samples collected into Streck tubes will be centrifuged and the supernatant stored frozen for the estimation of circulating tumour DNA.

The PAXgene-preserved blood sample will be stored for future analysis of germ-line DNA in relation to disease outcome, biological response (e.g. change in Ki67) to the aromatase inhibitor or tolerability of treatment.

Appendix 4 – WHO Performance Status

0	Normal activity
1	Strenuous activity restricted, can do light work
2	Up and about ≥ 50% waking hours, capable of self care
3	Confined to bed > 50 waking hours, limited self care
4	Confined to bed or chair, no self care, completely disabled

Appendix 5 - Patient information sheet and consent forms

For patient pathway A: patients should be offered either the POETIC generic information and consent form approved by Main REC, or generic patient information and consent forms approved by an ethics committee specifically for patients donating tissue to that tissue bank. Locally approved information and consent must be adequate to allow tissue to be used within POETIC. All patients who have donated tissue at diagnosis and invited to take part in POETIC must be offered the POETIC information sheet for Pathway A

For patient pathway B: patients must be offered the POETIC information sheet for Pathway B.

For patient at non-biological centre: patients must be offered the POETIC information sheet for non-biological centres.

Additional information and consent form for patients taking part in the POETIC study: additional information and consent form for patients taking part in the POETIC study version 1 - 09 May 2011 approved by Main REC. Patients consented to the POETIC patient information sheet and consent form Versions 1-3 (Version 1 – 12 March 2008, Version 2 – 01 August 2008, or Version 3 – 22 December 2009) approved by Main REC are/were asked to re-consent at their next visit to give permission for the extraction, storage and research on genetic material (DNA) in blood samples already donated.

Additional consent for late follow up blood samples and tissue at recurrence sample collection: patients should be invited to consent to the additional samples collection (late follow up blood and tissue at recurrence sample) ideally at their next routine or at an additional visit. Centres should contact participants in line with their local practice. Should the centres contact the patients initially via post, the letter provided to accompany the consent for additional samples collection may be used,

All patient information sheets and consent forms are provided as separate documents. Further guidance on gaining patient consent is provided in the Trial Guidance Notes.
